# Protective interaction of human phagocytic APC subsets with *Cryptococcus neoformans* induces genes associated with metabolism and antigen presentation

**DOI:** 10.3389/fimmu.2022.1054477

**Published:** 2022-11-15

**Authors:** Benjamin N. Nelson, Cheyenne S. Daugherty, Rachel R. Sharp, J. Leland Booth, Vineet I. Patel, Jordan P. Metcalf, Kenneth L. Jones, Karen L. Wozniak

**Affiliations:** ^1^ Department of Microbiology and Molecular Genetics, Oklahoma State University, Stillwater, OK, United States; ^2^ Harold Hamm Diabetes Center, University of Oklahoma Health Sciences Center, Oklahoma City, OK, United States; ^3^ Department of Medicine, Pulmonary, Critical Care & Sleep Medicine, University of Oklahoma Health Sciences Center, Oklahoma City, OK, United States; ^4^ Department of Cell Biology, University of Oklahoma Health Sciences Center, Oklahoma City, OK, United States; ^5^ Department of Microbiology and Immunology, University of Oklahoma Health Sciences Center, Oklahoma City, OK, United States; ^6^ Veterans Affairs Medical Center, Oklahoma City, OK, United States

**Keywords:** *Cryptococcus*, pulmonary phagocytes, macrophage subsets, dendritic cell subsets, pulmonary innate immunity, metabolism, antigen presentation

## Abstract

Cryptococcal meningitis is the most common cause of meningitis among HIV/AIDS patients in sub-Saharan Africa, and worldwide causes over 223,000 cases leading to more than 181,000 annual deaths. Usually, the fungus gets inhaled into the lungs where the initial interactions occur with pulmonary phagocytes such as dendritic cells and macrophages. Following phagocytosis, the pathogen can be killed or can replicate intracellularly. Previous studies in mice showed that different subsets of these innate immune cells can either be antifungal or permissive for intracellular fungal growth. Our studies tested phagocytic antigen-presenting cell (APC) subsets from the human lung against *C. neoformans*. Human bronchoalveolar lavage was processed for phagocytic APCs and incubated with *C. neoformans* for two hours to analyze the initial interactions and fate of the fungus, living or killed. Results showed all subsets (3 macrophage and 3 dendritic cell subsets) interacted with the fungus, and both living and killed morphologies were discernable within the subsets using imaging flow cytometry. Single cell RNA-seq identified several different clusters of cells which more closely related to interactions with *C. neoformans* and its protective capacity against the pathogen rather than discrete cellular subsets. Differential gene expression analyses identified several changes in the innate immune cell’s transcriptome as it kills the fungus including increases of TNF-α (*TNF*) and the switch to using fatty acid metabolism by upregulation of the gene *FABP4*. Also, increases of TNF-α correlated to cryptococcal interactions and uptake. Together, these analyses implicated signaling networks that regulate expression of many different genes – both metabolic and immune - as certain clusters of cells mount a protective response and kill the pathogen. Future studies will examine these genes and networks to understand the exact mechanism(s) these phagocytic APC subsets use to kill *C. neoformans* in order to develop immunotherapeutic strategies to combat this deadly disease.

## Introduction

*Cryptococcus neoformans* is an opportunistic fungal pathogen that causes pneumonia and fatal meningitis in immunocompromised individuals (1, 2). This fungus has an environmental niche that includes decayed wood and pigeon droppings. Humans frequently encounter either their yeast cells or desiccated spores which are then inhaled into the lungs (3, 4). The polysaccharide capsule surrounding the fungus allows it to ward off many first line innate immune defenses of the lung, but cell-mediated immunity (CMI), provided by Th1-type CD4^+^ T cells, is usually sufficient for clearance of the pathogen before any symptoms arise (2, 5, 6). However, in situations where the person has a suppressed immune system, such as during immunosuppressive therapy for organ transplants or HIV/AIDS, *C. neoformans* can survive and begin to replicate within the lung. Once established, pulmonary cryptococcosis presents clinically as pneumonia (7, 8). If left untreated, *C. neoformans* can traffic to the brain, presumably inside macrophages, causing highly fatal cryptococcal meningitis (9, 10). Cases of cryptococcal meningitis are highly associated with AIDS, which has led the CDC to name it as an AIDS-defining illness (11, 12). Evidence for this was demonstrated in an analysis of the 2014 Joint UN Programme on HIV and AIDS which estimated 223,100 yearly cases of these co-infections with a vast majority (73%) occurring within sub-Saharan Africa (13). These cases are also frequently lethal with mortality rates exceeding 80% worldwide. These numbers are unacceptably high, and reduction of the disease burden among affected individuals is paramount.

After inhalation, *C. neoformans* interacts with pulmonary innate immune cells including macrophages and dendritic cells (DCs) (14, 15). Effective immune responses will result in either phagocytosis and destruction of the fungal cell or sequestration and development of a cryptococcal granuloma (16). However, in immunocompromised individuals, killing does not always happen post-phagocytosis, and instead the pathogen will survive and begin to replicate within host macrophages (10, 17). From this point, it can travel throughout the rest of the body intracellularly *via* the bloodstream all while effectively evading the immune system (18). Normally, the blood brain barrier (BBB) will prevent pathogens or other unwanted substances from entering the central nervous system (CNS), however, *C. neoformans* has developed ways of crossing this obstacle either by direct transcellular migration of the fungus across the BBB or intracellularly within a trafficking immune cell (19–22). After traveling to the brain inside a pulmonary phagocyte, *C. neoformans* can exit the cell either by lytic or non-lytic means and cause infection and damage to the brain tissue (23, 24).

Cryptococcal interactions with innate immune cells have been studied extensively in the context of cell origin, lineage, and host condition. The first-line defenders in the pulmonary cavity to recognize *C. neoformans* are tissue resident macrophages and DCs (25). In both mice and humans, these innate phagocytes can kill the pathogen through both oxidative and non-oxidative means (26–29). And while both immune cell types are antigen-presenting cells (APCs), DCs are the primary antigen-presenting cells to present *Cryptococcus* antigens to naive T cells to initiate the CMI that is necessary in eradicating the disease (30). Following infection, neutrophils, another innate phagocyte, can infiltrate into the pulmonary tissue. Although *C. neoformans* can be phagocytosed by neutrophils, depletion of these cells in murine models leads to increased clearance of *C. neoformans*, suggesting that neutrophils may be detrimental (31–33). For cryptococcal clearance, the adaptive immune system relies on a Th1 and Th17 profile while Th2 cytokines correlate with fungal dissemination (7). Previous studies have shown that IFN-γ, IL-12, IL-17A, and IL-23 are beneficial for host survival and IL-13 and IL-33 lead to exacerbation of disease (34–37). Despite this, there is also evidence that depending on the activation phenotype, macrophages can either destroy the pathogen or harbor it intracellularly allowing for persistence of the fungus. Major factors of this include the polarization of the macrophage to the antifungal M1 (by cytokines IFN-γ and IL-12) or more permissive M2 phenotype (by cytokines IL-4, IL-5, and IL-13) (10, 38, 39). However, polarization of the macrophage does not occur in the naïve macrophages in the early preliminary stages of cryptococcosis, and there may be other factors including cell origin and subsets that can account for the differences in disease progression.

Current methods of treating cryptococcosis and cryptococcal meningitis include a variety of antifungal drugs including fluconazole, flucytosine, and amphotericin B (40–42). The gold standard for treatment of this disease includes a combination therapy of amphotericin B with flucytosine (43). However, due to high cost, lack of availability, and oversight for the compliance of the treatment regime in the most needed areas, fluconazole monotherapy is the usual treatment method (44). Complicating these problems, many of these antifungal drugs target ergosterol, the fungal analogue of cholesterol, and is often associated with toxicity to host cells at recommended doses (45). The combination of drug-mediated host cell toxicity, lack of antifungal drug development, and emerging resistance to current therapies, make it clear that there is need for additional treatment methods (46, 47). Fortunately, there has been recent work on improving the immune response to combat *C. neoformans* through the use of immunotherapeutics. These treatments include a variety of ways to enhance the body’s intrinsic immune systems to better recognize and eliminate the fungus. Options include the use of cryptococcal proteins as antigens to initiate protection through CMI (48, 49). All of these treatments must strike a balance between the protective Th1 responses and excessive inflammation that could worsen disease outcomes (50). In a mouse model of cryptococcal infection, adjunctive IFN-γ increases the antifungal effects of amphotericin B both *in vivo* and *in vitro* (51, 52). Despite the extensive work in this model, there is limited data directly applicable to human disease. However, these limited studies have shown that levels of IFN-γ are associated with protection in patients and that immunotherapies with adjunctive IFN-γ are well tolerated in humans and can lead to positive outcomes (53–55). The exact mechanisms whereby this therapy optimizes immune responses, specifically in first-line defenders such as lung macrophages and DCs is unknown.

Recently, subsets of macrophages and DCs of both human and mouse lungs have been characterized (25), and their lineages have been defined in recent years (56, 57). In the pulmonary airways and tissues of humans, macrophages have been historically divided into alveolar macrophages (AMs), which are in the alveolar cavity, and interstitial macrophages (IMs), which exist in the interstitium (58). In addition to differences in surface marker expression, there are differences of inflammatory capacity and phagocytic index between the subsets (59). DCs are similarly subdivided based on their surface markers, expression of pattern recognition receptors, and their ability to stimulate T cell proliferation (60). Due to these differences of lineages between tissue resident cells and circulating cells, it is important to isolate and investigate specifically the cell subset(s) *C. neoformans* comes into contact within the lungs (61–64) and the fate of the cryptococcal cells following uptake. Previous work has shown that there are indeed differences in the fate of this fungus (and other pathogens) depending on which subset it encounters. *Mycobacterium tuberculosis* (Mtb), another pulmonary pathogen which has a similar disease progression as *C. neoformans*, has differential interactions with macrophage subsets (65, 66). Specifically, murine IMs kill Mtb with a glycolytic metabolic profile while AMs allow for increased bacterial replication and are transcriptionally committed to fatty acid oxidation (67, 68). Additionally, our lab has shown differences in murine macrophage and DC subset responses to *C. neoformans*. Ly6c^-^ monocyte-like macrophages significantly inhibited the growth of *C. neoformans* while the CD11b^+^ conventional DCs significantly enhanced fungal growth (69). Between these two subsets, there were over 2,500 significantly differentially regulated genes, but the most prominent was differential regulation of MHC-I genes, with these being significantly upregulated in the antifungal Ly6c^-^ monocyte-like macrophage subset and significantly down-regulated in the permissive CD11b^+^ DC subset. In addition, metabolic genes – specifically those in the cytochrome P450 family (such as *CYP1B1*) were significantly up-regulated in the cells permissive for cryptococcal growth – the CD11b^+^ DCs. While these studies have been performed using the mouse models, it is important to investigate primary cells of human origin. Recently, six human pulmonary macrophage and DC subsets were consistently observed and profiled, including alveolar macrophages, CD14^-^ macrophages, CD14^+^ macrophages, CD207^+^ DCs, CD14^-^ DCs, and CD14^+^ DCs (70). These subsets differed in their ability to internalize a variety of bacterial species. All the subsets were more effective at internalizing the Gram-positives *S. aureus* and *B. anthracis* when compared to the Gram-negative *E. coli*. Furthermore, the human AMs and CD14^+^ DC and CD14^+^ macrophage subsets were more efficient at uptake than the other subsets. While these findings are significant, there are currently no studies that investigate cryptococcosis with human pulmonary phagocytes.

In this study, we investigated the initial interactions of *C. neoformans* with resident human pulmonary innate phagocytic APCs and determined how each subset responds to coincubation with cryptococcal cells *ex vivo*. Due to the differential interactions found in previously published studies, we hypothesized that the human subsets would have similar differential phenotypic patterns of killing versus permissive growth of the fungus as was found in the murine model (69). Human bronchial alveolar lavage (BAL) samples were enriched for phagocytic APCs and incubated with *C. neoformans* to determine uptake, killing ability, and transcriptional response. With these studies, we aimed to uncover the differential activity of human pulmonary macrophage and DC subsets and understand the genes and signaling pathways involved in these responses.

## Methods

### Reagents and media

Unless stated otherwise, chemical reagents of the highest quality were obtained from Sigma-Aldrich (St. Louis, MO) and tissue culture media of the Gibco brand and plasticware were both purchased from Thermo Fisher Scientific (Waltham, MA). R10 media is defined as RPMI 1640 supplemented with 10% heat-inactivated fetal bovine serum (FBS), 2 mM L-glutamine, 50 mM 2-mercaptoenthanol, 100 U penicillin/ml, and 100 μg streptomycin/ml, filter-sterilized through a 0.22µm filter. For the use of the Dead Cell Removal Kit (Miltenyi Biotech Inc., Bergisch Gladbach, Germany), 1X Binding Buffer was prepared by diluting 20X Binding Buffer Stock solution with double-distilled water. The FACS buffer used in this study was phosphate-buffered saline (PBS) + 2% FBS, filter-sterilized through a 0.22µm filter.

### Culture of *Cryptococcus neoformans*

*Cryptococcus neoformans* strains H99 (serotype A, mating type α) and mCherry producing strain JLCN920 (serotype A, KN99 mating type α), a kind gift of Dr. Jennifer Lodge (Duke University, Durham, NC), were recovered from 15% glycerol stocks stored at -80°C and were cultured for 18h at 30°C with shaking in yeast extract-peptone-dextrose (YPD) broth (BD Difco; Franklin Lakes, NJ) and collected by centrifugation. Organisms were washed three times with sterile PBS, and viable yeast cells were quantified using trypan blue dye exclusion in a hemocytometer. Cryptococcal cells were resuspended in appropriate medium at the concentration needed for each experiment.

### Human bronchoalveolar lavage sample processing

Isolation and preservation of lavaged lung cells was performed as previously described (70, 71). Briefly, whole human donor lungs were obtained through LifeShare of Oklahoma (Oklahoma City, OK, USA; http://www.lifeshareoklahoma.org) and the International Institute for the Advancement of Medicine (IIAM, Edison, NJ, USA; http://www.iiam.org). Lungs were deemed nontransplantable for reasons such as histocompatibility mismatches, lung size, uncertain drug usage, or prior incarceration. Our criteria for lung acceptance included: 18–70 years of age, no history of smoking tobacco or nonsmoking a minimum of 2 years, no history of lung disease, noncardiac death, a PaO2/FiO2 ratio > 200, and normal to minimal atelectasis based on chest x-ray results, with no evidence of intercurrent infection. The donor demographics for the lungs used in this study can be found in **Table S1**. Lungs were harvested and transported as per transplant protocols. Upon arrival at the Metcalf laboratory at OUHSC, Wisconsin solution and residual blood was washed from the vasculature using sterile physiological saline (0.9% w/v). Saline was pumped at low pressure (∼20 cm water) into the main bronchus to produce visible swelling of lobes. The resultant BAL collections were pooled, and cells were concentrated by centrifugation at 300 x *g* for 10 minutes, resuspended to 1 × 10^7^ cells/ml in freeze medium (40% RPMI 1640, 50% FBS, and 10% DMSO) (72), frozen in 2 ml aliquots at a rate of ∼1°C/min at -80°C, and stored in liquid nitrogen vapor at -190°C. For experiments, aliquots were thawed quickly at 37°C in a Precision™ GP-10 water bath (Thermo Fisher Scientific) and allowed to settle in R10 media for 10 minutes at room temperature (RT) followed by centrifugation at 1000 x *g* for 5 minutes. Dead cells were removed from collected cells with the Dead Cell Removal Kit (Miltenyi Biotech Inc.) as per manufacturer’s protocol with the following changes: 5X recommend amount of Dead Cell Removal Microbeads (500 μl per 10^7^ cells) were used in order to properly label excessive dead cells and separation occurred within LS columns (Miltenyi Biotech Inc.). Viable cells were quantified using trypan blue exclusion dye in a hemacytometer.

### Active cryptococcosis detection

Presence of active cryptococcal infection was determined using the Cryptococcal Antigen Lateral Flow Assay (CrAg^®^ LFA; IMMY, Inc., Norman, OK) as per manufacturer’s instructions.

### Cryptococcal uptake by standard flow cytometry

Processed human BAL cells were incubated with fluorescent mCherry expressing *C. neoformans* strain JLCN920 at a 1:1 ratio (1 × 10^6^ cells each) with 1 μg/ml anti-GXM mAb F12D2 (kind gift of Dr. Tom Kozel, University of Nevada Reno, Reno, NV) at 37°C, 5% CO_2_ for 2h in 100 μl of R10 media. Following incubation, samples were resuspended in 100 μl of FACS buffer and stained with the CD45 leukocyte marker, a lineage cocktail (CD3, CD19, CD20, and CD56), a viability dye, calcofluor white (CFW), and subset markers (specific markers and fluorophores used are shown in **Table S2**) for 30 minutes at 4°C. Samples were washed three times and then fixed with 2% formaldehyde and subsequently analyzed on a Novocyte 3000 flow cytometer (Agilent Technologies, Inc., Santa Clara, CA) with data analyzed using NovoExpress software (Agilent Technologies, Inc.). Gating scheme for detection of populations and associations was based on previously published research (70) and are shown in **Figure S2**. Gates were determined using controls including live/dead stain, unstained cells, single color, and isotype control samples. To calculate percent uptake, the total number of cells that were double-positive (positive for subset markers and positive for mCherry) were divided by the total number of that subset, then multiplied by 100. Percent was then plotted for each individual patient.

### Cryptococcal morphology by imaging flow cytometry

Processed human BAL cells were enriched for phagocytic populations by negative selection of CD3, CD19, CD20, and CD56 microbeads and magnetic separation (Miltenyi Biotech Inc.) as per manufacturer’s instructions. Phagocytic populations were then incubated with mCherry expressing *C. neoformans* strain JLCN920 at a 1:1 ratio (1 × 10^6^ cells each) with 1 μg/ml anti-GXM mAb F12D2 at 37°C, 5% CO_2_ for 2h in 100 μl of R10 media. Following incubation, samples were separated into half (one for each of the two different staining schemes needed for analysis), resuspended in 100 μl of FACS buffer and stained with leukocyte and subset markers (specific markers and fluorophores used are shown in **Table S2**) for 30 minutes at 4°C. Samples were washed three times and then fixed with 1% paraformaldehyde and subsequently analyzed on an Amnis^®^ ImageStream^®^X Mk II Imaging Flow Cytometer (Luminex Corporation, Austin, TX) with data analyzed using IDEAS^®^ 6.2 software (Luminex Corporation). Gating scheme for detection of populations and internalization is shown in **Figure S3**. Gates were determined using single color controls with UltraComp eBeads™ Compensation Beads (Thermo Fisher Scientific). Quantification of fungal killing results was conducted by examining up to 100 morphologies of internalized cryptococcal cells for each subset. Previous research has indicated that budding and single round cryptococcal cells are viable and condensed cells and debris are nonviable cells (73).

### Cryptococcal uptake for single cell RNA sequencing

Processed human BAL cells were enriched for phagocytic populations as previously stated above and incubated with *C. neoformans* strain H99 at a 1:1 ratio (1 × 10^6^ cells each) with 1 μg/ml anti-GXM mAb F12D2 at 37°C, 5% CO_2_ for 2h in 100 μl of R10 media. Controls were phagocytic populations from the same human samples in media alone incubated under the same conditions. Samples were then washed once and then resuspended with PBS + 2% BSA. Libraries were made of pairs of controls and co-incubated sets using the 10X Chromium Controller and the Chromium Next Gem Single Cell 3’ GEM, Library, and Gel Bead Kit v3.1 (10X Genomics Inc., Pleasanton, CA) and sequenced on a Novaseq series sequencer (Illumina Inc., San Diego, CA) as per manufacturer’s protocols.

### Identification of clusters of macrophages and DCs

Single cell analysis and cluster identification was performed on 1,654 – 16,903 captured cells per sample using the 10X Chromium single cell system (10X Genomics Inc.) at the University of Oklahoma Health Sciences Campus Genomics Core Facility and sequenced each sample to a read depth of 95-164K reads/cell, yielding 85-92% sequence saturation. Read mapping and expression quantification were performed using a combination of the 10X Cell Ranger pipeline (10X Genomics Inc.) and custom Seurat analytic scripts (74). Briefly, single-cell reads were mapped to the human genome (GRCH38) and assigned to genes using the standard Cell Ranger pipeline. Normalized gene expression was then used to produce a UMAP plot that provided cell clusters based on similarity of gene expression. Once cells were assigned to a cluster, custom Seurat scripts were used to statistically derive the gene expression differences within and between cell clusters using *t*-tests in Seurat. Trajectory analysis was then conducted using Monocle (75). To be statistically similar across the study, the Monocle trajectories were used to guide specific trajectory specific *t*-tests within Seurat. From these, pathway analyses were performed using Ingenuity Pathway Analysis (IPA, QIAGEN, Hilden, Germany) on the differential transcriptional profiles seen in the cell clusters and trajectory groups.

### TNF-α intracellular imaging flow cytometry

Processed human BAL cells were enriched for phagocytic populations as previously stated and incubated with *C. neoformans* strain JLCN920 at a 1:1 ratio (1 × 10^6^ cells each) with 1 μg/ml anti-GXM mAb F12D2 at 37°C, 5% CO_2_ for 2h in 100 μl of R10 media. BD GolgiStop™ and BD GolgiPlug™ (BD Biosciences, Franklin Lakes, NJ) were used, as per manufacturer’s protocol, to prevent cytokine secretion. Following incubation, samples were separated into half (one for each of the two different staining schemes), resuspended in 100 μl of FACS buffer and stained with either TNF-α panel #1 or #2 (**Table S2**). Extracellular staining was performed as previously stated for 30 minutes at 4°C. Following extracellular staining, cells were fixed with 2% formaldehyde for 10 minutes at room temperature and permeabilized with Intracellular Staining Perm Wash Buffer (BioLegend^®^, San Diego, CA) for 10 minutes at room temperature. Cells were stained with intracellular markers (IL-1β, CCL3, CXCL8 and TNF-α), washed, and fixed with 1% paraformaldehyde. Samples were analyzed on an Amnis^®^ ImageStream^®^X Mk II Imaging Flow Cytometer (Luminex Corporation) with data analyzed using IDEAS^®^ 6.2 software (Luminex Corporation). Gating scheme for detection of populations and internalization are shown in **Figure S6**. Gates were determined using single color controls with UltraComp eBeads™ Compensation Beads (Thermo Fisher Scientific).

### Statistical analysis

All statistical analyses were conducted using GraphPad Prism version 5.00 for Windows (GraphPad Software, San Diego, CA) unless noted otherwise. Significant differences were defined as *p* < 0.05 and α = 0.05 unless noted otherwise. The student’s unpaired two-tailed *t*-test was performed to calculate level of significance among MFI means of CXCL8, CCL3, IL-1β, and TNF-α between phagocytic APCs which contained *C. neoformans* versus cells without the fungus. One-way ANOVA with Tukey’s Multiple Comparison Test was performed to compare medians of human phagocytic APC subsets, their uptake of *C. neoformans*, and percent cryptococcal killing of each subset. Linear regression was performed to calculate the line of best fit in correlations of JLCN920 fluorescence intensities to antibody intensities for markers CXCL8, CCL3, IL-1β, and TNF-α. scRNA-seq analysis, cluster identification, and trajectory analysis was performed with a combination of Cell Ranger pipeline, Seurat, Monocle, and custom R scripts.

## Results

### Phagocytic APC subsets were identified in human bronchoalveolar samples and interact with *C. neoformans*

**Figure 1:**
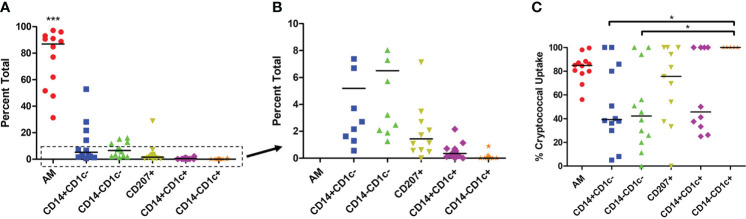
Percent Population of Each Subset Identified and Interact with *C neoformans*. Processed human BAL cells were incubated with fluorescent mCherry expressing *C. neoformans* strain JLCN920 for 2h and then stained with fluorescent antibodies for flow cytometric analysis. **(A)** Representation of each phagocytic APC subset shown as a percentage of total population indicated by surface markers. **(B)** Inset from panel **(A)** showing relative abundance of remaining low number subsets. **(C)** Percentage of uptake by each phagocytic APC subset of *C. neoformans* indicated as positive for mCherry. Data points shown are individual samples with black bars as median of the results of 12 samples (n=12), with each sample performed in duplicate. One-way ANOVA with Tukey’s Multiple Comparison Test was performed to compare each column against all others. *** indicates significantly different means of AM subset to all others (*p* < 0.0001) and * represents significantly different means of indicated macrophage subset to DC subset CD14^-^CD1c^+^ (*p* < 0.05).

We sought to understand the differences in cryptococcal killing by macrophage and DC subsets (15, 18, 38, 69, 76), and, therefore, chose to follow up on a study that identified subsets of phagocytic APCs within the human lung (70). To avoid biases and remove the possibility of pre-activation that come with an active cryptococcal infection, we used a commercially available detection kit (CrAg^®^ LFA) to identify *Cryptococcus*-positive samples. None of the samples used in this study tested positive for infection, so we could, therefore, assume the fungal cells were not activated (representative image of results in **Figure S1** and results in **Table S3**). We next removed dead cells from the human BAL samples before incubating live cells with a fluorescent mCherry expressing *C. neoformans* strain JLCN920. Following a 2-hour incubation to allow for cryptococcal interaction and possible uptake, the phagocytic APCs were stained with fluorescent markers and analyzed by flow cytometry (gating scheme shown in **Figure S2**). **Figure 1A** shows the percent total of each of the previously identified subsets, and, in accordance with previous findings, the first three groups (AM, CD14^+^CD1c^-^, CD14^-^CD1c^-^) are macrophages while the last three groups (CD207^+^, CD14^+^CD1c^+^, CD14^-^CD1c^+^) are DCs (70). Alveolar macrophages (AMs) comprised over 85% of the total population, but despite being very low in total numbers, the other five subsets were identified within the samples (**Figure 1B**), with the occasional subset being undetectable (recorded as 0%). Next, we identified the percent that each of the detected subsets phagocytosed the fluorescent *C. neoformans*. Using calcofluor white (CFW) post**-**incubation, we were able to detect which fungi were external or on the surface of the phagocytic APCs and exclude those from the findings through the gating scheme. Results indicate that all six subsets interacted and phagocytosed the fungus to some degree (**Figure 1C**). However, two macrophage subsets (CD14^+^CD1c^-^ and CD14^+^CD1c^-^) had significantly lower uptake when compared to one of the dendritic cell subsets (CD14^-^CD1c^+^).

### Subsets of phagocytic APCs show differential killing of *C. neoformans*

**Figure 2:**
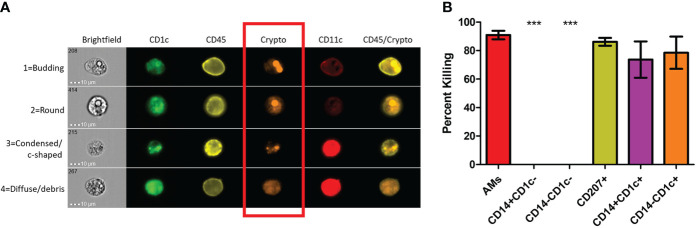
Subsets of Phagocytic APCs Show Differential Killing of *C neoformans*. Processed human BAL cells were enriched for phagocytic APCs and incubated with fluorescent mCherry expressing *C neoformans* strain JLCN920 for 2h and then stained with fluorescent antibodies for imaging flow cytometric analysis. **(A)** Representative images of internalized *C neoformans* to determine fate of the fungus. Budding and round fungal cells were considered as living while condensed and debris were deemed at dead cells (red box). **(B)** Percent killing of *C neoformans* by phagocytic APC subsets. Up to 100 images of internalized cryptococcal cells by each subset were analyzed according to panel **(A)**. 4 of the 6 subsets showed substantial killing (>70%) of internalized fungal cells. Macrophage subsets CD14^+^CD1c^-^ and CD14^-^CD1c^-^ allowed live fungal cells to persist internally in all analyzed images. Data shown are means ± standard errors of the means (SEM) of the results of 2 independent experiments using 12 independent BAL samples (n=12). One-way ANOVA with Tukey’s Multiple Comparison Test was performed to compare each column against all others. *** indicates significantly different means from killing subsets (*p* < 0.0001).

Previous studies have shown that dead or dying *C. neoformans* take on a deformed morphology (c-shaped or condensed) rather than their usual round or budding appearance while alive (73). Using morphology as a proxy for cryptococcal fitness, we examined several of these internalized fungal cells within each subset of phagocytic APCs. Prior to use, the human BAL cells were enriched for phagocytic APCs by removal of lineage-positive cells (CD3, CD19, CD20, CD56). Then cells were incubated with the mCherry-producing cryptococcal strain and incubated for 2 hours. Using imaging flow cytometry (gating scheme shown in **Figure S3**), we were able to discern the different morphologies of internalized cryptococcal cells while also identifying the phagocytic subset. Fungal fitness was scored according to their morphologies and examples of the system used in this study are shown in **Figure 2A**. By convention, cryptococcal cells which appeared either as round or budding are deemed alive while those that take on a more deformed shape are considered dead/dying (73). Cells that were positive for the fungal marker (fluorescent in the mCherry channel) but showed no discernable fungal shape but rather a diffusive staining were also determined to be dead. All the captured images (up to 100) for each of the subsets were quantified and graphed according to cryptococcal fate (**Figure 2B**). Results show that one of the macrophage subsets (AMs) and all the DC subsets were able to kill the fungus with high percentages of antifungal activity (>70%). However, macrophage subsets CD14^+^CD1c^-^ and CD14^-^CD1c^-^ were unable to kill internalized *C. neoformans* (0% dead/dying morphologies).

### ScRNA-seq identified different clusters of phagocytic APCs

**Figure 3:**
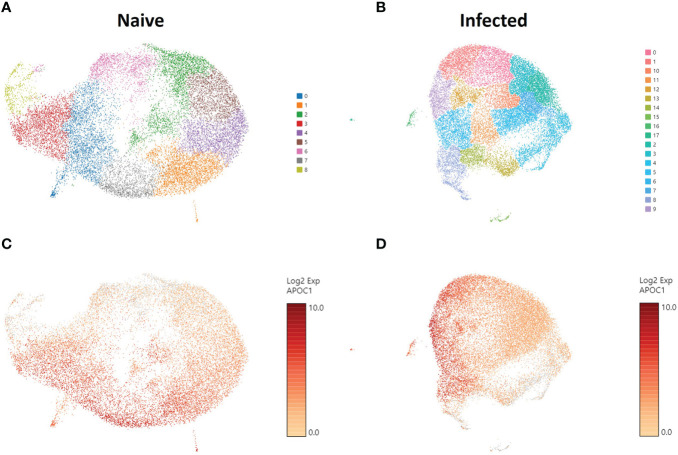
Phagocytic APCs Incubated with and without *C neoformans* Show Distinct Clustering of Cells which Are Not Correlated with Macrophage Identity. Processed human BAL cells were enriched for phagocytic APCs and incubated with or without *C neoformans* strain H99 for 2h and then processed for scRNA-seq. scRNA-seq analysis identified 9 and 18 discrete clusters among the naïve **(A)** and infected **(B)** cohorts, respectively. Cells were also examined for the expression and distribution of *APOC1* (macrophage gene) for both naïve **(C)** and infected **(D)** cohorts. The distribution of macrophages did not follow the clustering scheme for either of the cohorts. Data shown are transcriptional regulation of shown genes for 18,958 (naïve) and 22,344 (infected) total cells for three individual experiments for each cohort (n=3).

To understand the gene expression in the phagocytic APC subsets that displayed different cryptococcal killing phenotype during the early stages of cryptococcal interaction, RNA-seq was performed to investigate their transcriptome. Once the samples are thawed, the life span of cells is extremely limited (~4 hours) and physical separation of the cells into discrete groups is not possible. Therefore, single cell RNA sequencing (scRNA-seq) was performed for the level of resolution needed to identify differential clusters prior to this time (77, 78). Human BAL samples were enriched for phagocytic APCs by removal of lineage-positive cells (CD3, CD19, CD20, CD56), and then incubated with *C. neoformans* for two hours to allow for initial interaction or uptake. Control cells from each human sample were incubated under the same conditions without the pathogen. Analysis revealed 9 individual clusters among 18,958 total cells for the control cohort (naïve) and 18 clusters among 22,344 total cells for cells incubated with *C. neoformans* (infected) (**Figures 3A, B**). We initially investigated the distribution of macrophages among the given clusters by use of the macrophage gene *APOC1* (79). Projections of this gene onto the cluster map space revealed no pattern of clustering in either the naïve or infected cohorts (**Figures 3C, D**). Previously, identification of each phagocytic APC subset was accomplished using surface markers, and the transcriptional profile for naïve cells of each subset was determined (70). Using a single gene from the previous transcriptional profile to identify each subset, we identified cells from each subset in our samples; however, distribution of each subset also did not follow computational clustering, so we were unable to cluster the cells according to subset for either naïve or infected cohorts (**Figures S4A–L**).

### Trajectory analysis splits clusters into distinct groups with different phenotypes

**Figure 4:**
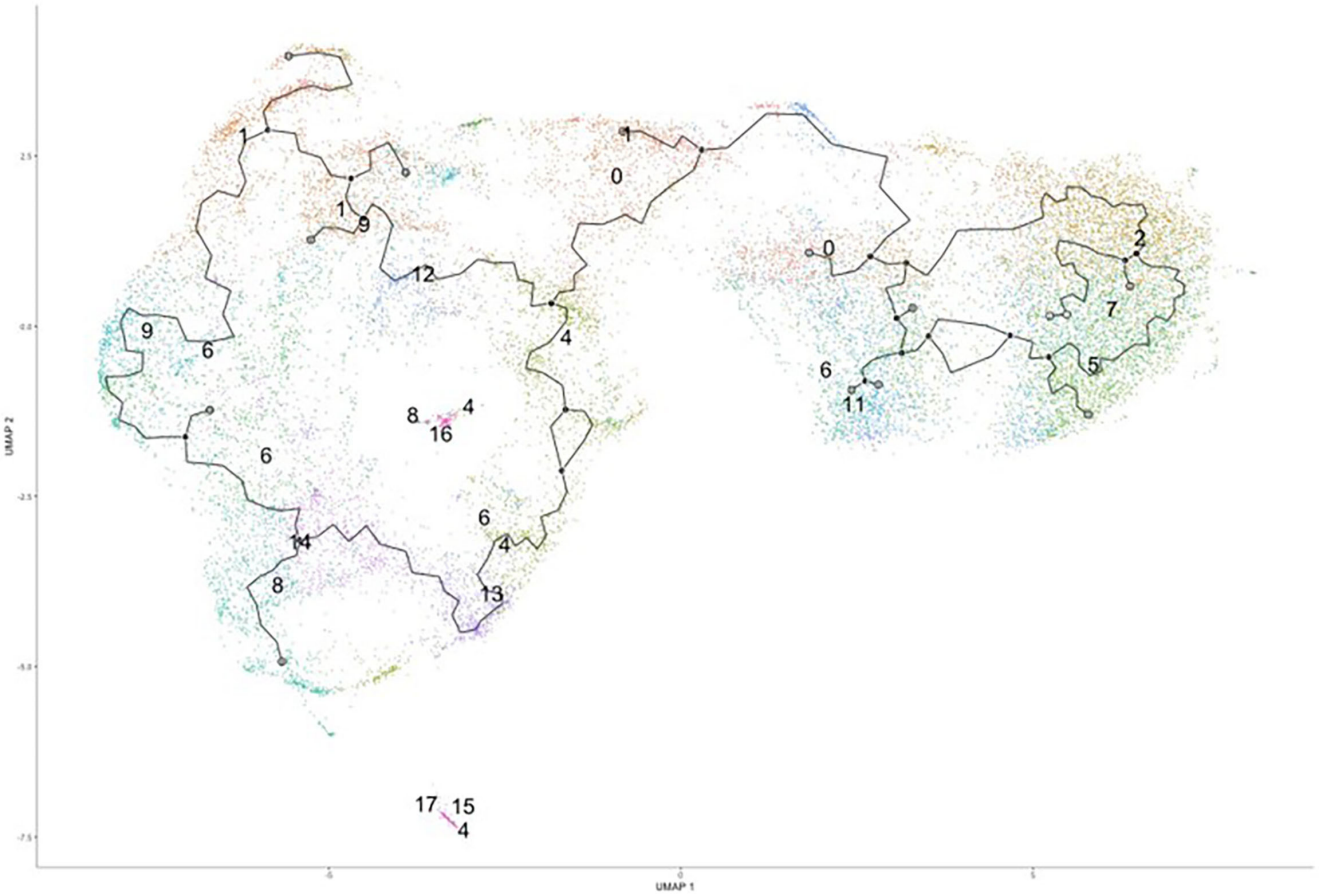
Trajectory Analysis Shows Divergent of Clusters into Two Main Groups. Processed human BAL cells were enriched for phagocytic APCs and incubated with *C. neoformans* strain H99 for 2h and processed for scRNA-seq. Uniform manifold approximation and projection (UMAP) plot was created from the 18 clusters of the infected cohort. From the initial cluster of #0, there is an immediate split of the clusters which follow clusters #1/9/6/12 towards to the left and clusters #2/7/5/3 to the right. Data shown are the 18 Seurat clusters for 22,344 total cells from the infected cohort for three individual experiments for each cohort (n=3).

**Figure 5:**
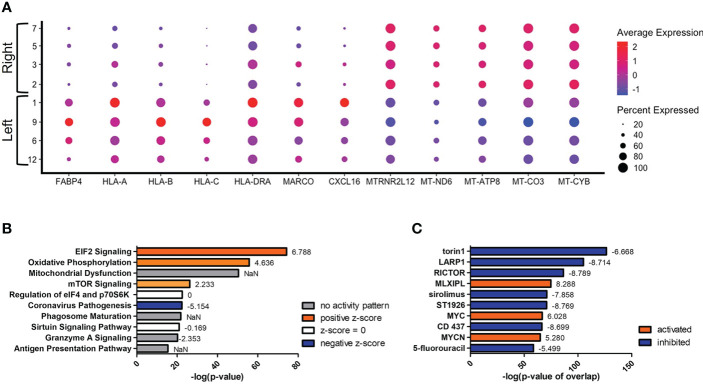
Upregulation of Antifungal Genes in Left Side Clusters #1/9/6/12. Processed human BAL cells were enriched for phagocytic APCs and incubated with *C neoformans* strain H99 for 2h and processed for scRNA-seq. Genes differentially expressed in clusters #1/9/6/12 when compared to clusters #2/7/5/3 were analyzed by IPA. **(A)** Differential expression of select genes in clusters #1/9/6/12 (left) and #2/7/5/3 (right). Top 10 significantly regulated canonical pathways **(B)** and predicted upstream regulators **(C)** as ordered by *p*-value or *p*-value of overlap. To the right of each bar is the statistical z-score for each pathway or upstream regulator with positive and negative being increased and decreased, respectively (NaN = not a number, no activity pattern). Data shown are combined analysis of cells from three individual experiments (n=3).

Since we were unable to isolate individual subsets, we sought to instead focus our efforts on the phenotypic outcomes and cellular processes rather than the inherent subset and their molecular differences. Since there are double the number of clusters in the infected cohort when compared to the naïve using the same standard for cluster discrimination and similar cell numbers, *C. neoformans* may have influenced the number of discrete clusters by causing a transcriptional change in some of the immune cells. With this evidence of a change in gene expression, a trajectory analysis was necessary to understand the full picture of the discrete clusters and how they compared to one another. Shown in **Figure 4** are the trajectory analyses conducted by R package Monocle on the 18 clusters identified in the infected cohort. Using cluster #0 as the starting point, there is a stark divergence between cells on the left and right side of the plot (**Figure 4**). Clusters 1 ➔ 9 ➔ 12 ➔ 6 follow along one transcriptional evolution (left) while clusters 3 ➔ 2 ➔ 7 ➔ 5 follow another progression (right). This departure between the paths is driven mainly by mitochondrial genes (including *MTRNR2L12* [*p* ≈ 0], *MT-ND6* [*p* ≈ 0], *MT-ATP8* [*p* = 4.67 × 10^-237^], *MT-CO3* [*p* ≈ 0], *MT-CYB* [*p* ≈ 0]) on the right and lipid metabolism genes (such as *FABP4* [*p* ≈ 0]) coupled with immune recognition genes (such as *MARCO* [*p* = 2.90 × 10^-185^] and *CXCL16* [*p* ≈ 0]) and antigen processing genes (*HLA-A* [*p* = 5.58 × 10^-290^], *-B* [*p* ≈ 0], *-C* [*p* ≈ 0], *HLA-DRA* [*p* = 1.72 × 10^-188^]) on the left (**Figure 5A**). A complete list of differentially expressed genes can be found in **Table S4**. The canonical pathways and predicted upstream regulators were analyzed by Ingenuity Pathway Analysis (IPA, QIAGEN) and the most significantly affected are shown in **Figures 5B, C**. The most altered pathways were EIF2 signaling and oxidative phosphorylation, which indicates the cells are altering their protein products and metabolism (**Figure 5B**). Pathways such as phagosome maturation and antigen presentation are affected by differences in MHC class I (*HLA-A*, *-B*, *-C*) and class II (*HLA-DR*) molecules along with the scavenger receptors *MARCO* and *CXCL16*. Most of the highest predicted upstream regulators involve mTOR (including torin-1, LARP1, and RICTOR) which can coordinate mitochondrial energy production (**Figure 5C**) (80). Taken together, these patterns indicate a cryptococcal killing phenotype for the left-side clusters.

**Figure 6:**
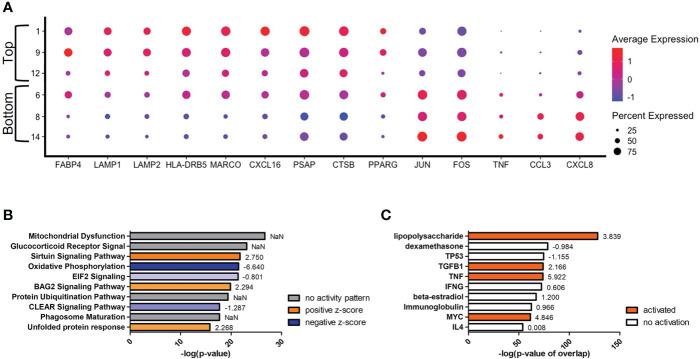
Transcriptome Evolution to Active State in Clusters #8/14. Processed human BAL cells were enriched for phagocytic APCs and incubated with *C neoformans* strain H99 for 2h and processed for scRNA-seq. Genes differentially expressed in clusters #8/14 when compared to clusters #1/9/12 were analyzed by IPA. **(A)** Differential expression of select genes in clusters #6/8/14 (bottom) and #1/9/12 (top). Top 10 significantly regulated canonical pathways **(B)** and predicted upstream regulators **(C)** as ordered by *p*-value or *p*-value of overlap. To the right of each bar is the statistical z-score for each pathway or upstream regulator with positive and negative being increased and decreased, respectively (NaN = not a number, no activity pattern). Data shown are combined cells from three individual experiments (n=3).

Secondary to the initial split in the trajectory plot, on the left side there are indications of a further evolution of transcriptomes from clusters #1/9/12 (top) to #8/14 (bottom) (**Figure 6**). The most differentially expressed genes in this trajectory are *CCL3* and *CXCL8*, both genes downstream of TNF-α and indicative of a protective response against *C. neoformans* (**Figure 6A**) (81, 82). These genes are upregulated in the lower clusters. A complete list of differentially expressed genes can be found in **Table S5**. Among the upper clusters, there were an increase in expression of genes that directly recognize cryptococcal cells such as the scavenger receptors *MARCO* (*p* ≈ 0) and *CXCL16* (*p* ≈ 0), stress sensor PPARγ (*p* = 9.93 × 10^-82^), the lysosomal indicators *LAMP1* (*p* = 3.62 × 10^-168^) and *LAMP2* (*p* = 6.43 × 10^-179^), genes that contribute to the antifungal activity of lysosomes (*CTSB* [*p* = 2.64 × 10^-187^] or cathepsin B; *PSAP* [*p* ≈ 0], the precursor to saposins A-D), and the antigen presentation MHC-II gene *HLA-DRB5* (*p* ≈ 0). All these were accompanied with an increase of gene expression indicating fatty acid metabolism (*FABP4* [*p* = 1.09 × 10^-281^]). As we move into the lower clusters, there is a switch from genes that deal directly with *C. neoformans* to genes involved in cytokine responses with increases in expression of the previously mentioned genes TNF-α (*TNF*) (*p* = 5.30 × 10^-75^), *CCL3* (*p* = 1.40 × 10^-241^), *CXCL8* (*p* ≈ 0) along with transcriptional factors *JUN* (*p* = 3.84 × 10^-312^) and *FOS* (*p* = 3.84 × 10^-159^). Again, these differentially regulated genes were analyzed by IPA and the top changes to canonical pathways and predicted upstream regulators are shown in **Figures 6B, C**. Similar to the left versus right analysis, the most differentially regulated pathway are general pathways that control many cellular functions including mitochondrial functions, glucocorticoid receptor signaling, and oxidative phosphorylation. However, pathways that control immune responses are also present in the form of the sirtuin signaling and CLEAR (coordinated lysosomal expression and regulation) signaling pathways (**Figure 6B**) (83, 84). Among the predicted upstream regulators, LPS and dexamethasone are the most significant and are linked to the expression of *FABP4* (**Figure 6C**). This is congruent to the actual expression of *FABP4* which is relatively high in the top clusters and tapers off in bottom clusters.

### Increases in TNF-α correlate with changes of cellular stress response and metabolism in early clusters

**Figure 7:**
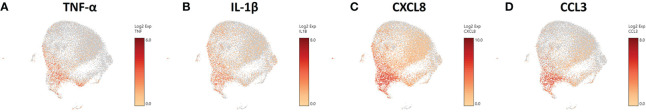
Phagocytic APCs Incubated with *C. neoformans* Are Differential in TNF-α and Genes Downstream of TNF-α. Processed human BAL cells were enriched for phagocytic APCs and incubated with *C neoformans* strain H99 for 2h and then processed for scRNA-seq. scRNA-seq analysis identified differential regulation of TNF-α (*TNF*) **(A)**, *IL-1β* **(B)**, *CXCL8* **(C)**, and *CCL3* **(D)** that occur primarily in cells in the bottom left portion of the plot. Data shown for are transcriptional regulation of shown genes for 22,344 total cells for three individual experiments (n=3).

With TNF-α known to provide a protective response against *C. neoformans* (81, 85) and coupled with our data on the increases of TNF-α gene expression in particular clusters, we focused our attention to the transcriptional effects that interaction with the fungus has on our phagocytic APCs. Examining the scRNA-seq data and the distribution of TNF-α gene expression in the infected cohort, we found that the cells up-regulating TNF-α (*TNF*) are in the lower left portion of the population (**Figure 7A**). This trend continues in genes downstream of TNF-α signaling as well, including *IL-1β*, *CXCL8*, and *CCL3* (**Figures 7B-D**), which each contribute to protection against *Cryptococcus* through feedback loops (86–88). TNF-α also manipulates macrophage activation and although this study focused on early time points and events that occur prior to macrophage activation (and in the absence of T cells or T cell-associated cytokines), activation to either the protective M1 or permissive M2 phenotypes as well as Th1/Th2-type cytokine production may also affect cryptococcal fate (89, 90). Within the clusters of the infected cohort, we found no transcriptional indication of macrophage activation to either M1 or a Th1-type phenotype (*IFN-γ*, *NOS2*, *IL-12A, IL-12B*) or M2 or a Th2-type phenotype (*IL-4*, *IL-10*, *IL-13*, *ARG1*) (**Figures S5A–H**). This information allows us to investigate the phenotypic differences caused by a cryptococcal infection as inherent differences of cellular processes among the cells of the samples as opposed to a generalized transcriptional skewing and activation of the inflammatory network.

**Figure 8:**
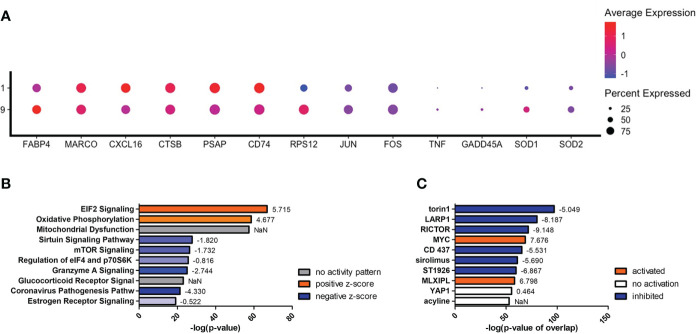
Differentially Expressed Genes in Cluster #9 Led to Changes in Cellular Stress Response and Metabolism. Processed human BAL cells were enriched for phagocytic APCs and incubated with *C neoformans* strain H99 for 2h and processed for scRNA-seq. Genes differentially expressed in cluster #9 when compared to cluster #1 were analyzed by IPA. **(A)** Differential expression of select genes in clusters #9 and #1. Top 10 significantly regulated canonical pathways **(B)** and predicted upstream regulators **(C)** as ordered by *p*-value or *p*-value of overlap. To the right of each bar is the statistical z-score for each pathway or upstream regulator with positive and negative being increased and decreased, respectively (NaN = not a number, no activity pattern). Data shown are combined cells from three individual experiments (n=3).

To understand what occurs when TNF-α is upregulated, we investigated clusters #1 and #9, which are among the early left side group and have differential TNF-α activation (*p* = 1.84 × 10^-11^). Compared to cluster #9, cells in cluster #1 display an increase in genes encoding for scavenger receptors (*MARCO* [p = 0.001] and *CXCL16* [*p* = 2.67 × 10^-95^]), cryptococcal killing machinery (*CTSB* [*p* = 2.23 × 10^-10^] and *PSAP* [*p* = 2.00 × 10^-130^]), and the MHC-II chaperone *CD74* (*p* = 6.39 × 10^-100^) (**Figure 8A**). As TNF-α becomes upregulated in cluster #9, the cells do not induce the aforementioned genes, but instead switch to more signaling pathway genes that include the stress sensor *GADD45α* (*p* = 1.60 × 10^-11^) and the transcription factors *JUN* (*p* = 5.32 × 10^-53^) and *FOS* (*p* = 2.41 × 10^-20^). This is evident in the most highly upregulated genes being ribosomal genes (like *RPS12* [*p* = 7.50 × 10^-305^]) and is accompanied by a utilization of fatty acid metabolism through *FABP4* (*p* = 1.46 × 10^-28^). Finally, superoxide dismutase genes (*SOD1* [*p* = 1.61 × 10^-27^] and *SOD2* [*p* = 2.71 × 10^-19^]) are also increased along with TNF-α (**Figure 8A**). A complete list of differentially expressed genes can be found in **Table S6**. Examining the most regulated canonical pathways and upstream regulators, many of the affected gene sets belong to cellular wide functions including EIF2 signaling, oxidative phosphorylation, and glucocorticoid receptor signaling as well as master regulators like torin-1, LARP1, RICTOR, and MYC (**Figures 8B, C**). These results show that the cells are significantly changing their proteome and metabolism to adapt to a change in stress.

### Differential staining of TNF-α markers indicates cryptococcal interaction

**Figure 9:**
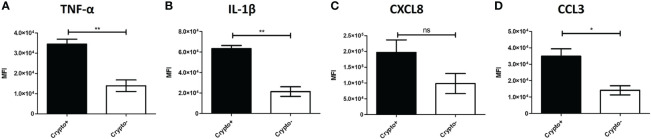
Phagocytic APCs Incubated with *C neoformans* Show Differential TNF-α and Downstream Protein Expression Following Fungal Interaction. Processed human BAL cells were enriched for phagocytic APCs and incubated with mCherry expressing *C neoformans* strain JLCN920 for 2h and then processed for imaging flow cytometric analysis. Analysis shows that increased presence of protein expression of TNF-α **(A)**, IL-1β **(B)**, and CCL3 **(D)** occur when interaction with cryptococcal cells was verified compared to non-interacting cells. CXCL8 **(C)** shows a similar but non-significant trend, as well. Data shown are mean fluorescent intensities (MFI) of individual viable CD45^+^ phagocytic APCs from three individual experiments (n=3). Two-tailed *t*-tests were performed to compare pairs of columns. * and ** indicates significantly different means of *p* < 0.05 and *p* < 0.0001, respectively.

To investigate these transcriptional differences that are accompanied with changes in TNF-α expression in the phagocytic APCs and how they relate with intracellular cryptococcal morphology, human BAL cells were again incubated with mCherry expressing *C. neoformans* strain JLCN920 followed by an examination of the intracellular cryptococcal morphology as it correlates with protein production of TNF-α (and downstream markers IL-1β, CXCL8, and CCL3) on an imaging flow cytometer (gating scheme shown in **Figure S6**). Cryptococcal morphology was again used and scored as described above to indicate the fate of the pathogen (**Figure S7**). We found that there were no significant differences of the fluorescent intensities for TNF-α or any associated downstream markers between living and killed intracellular cryptococcal cells (**Figures S8A–D**). However, a linear regression analysis showed a strong positive correlation between all intracellular cryptococcal cells (both living and killed) and these markers (**Figures S8E–H**). This led us to examine the differences of these markers in immune cells that have either interacted (high mCherry intensity) or not interacted (low mCherry intensity) with *C. neoformans*. Using the cutoff provided by the negative cryptococcal staining control, we found significant differences of staining intensities between phagocytic APCs that have interacted with the fungus versus those that have not in three (*p*-values = TNF-α: 0.0053, IL-1β: 0.0017, CCL3: 0.0170) of the four markers, with the fourth (CXCL8, *p*-value = 0.1242) trending in the same direction (**Figures 9A–D**). These data show that TNF-α, CCL3, IL-1β, and CXCL8 protein levels are correlated with cryptococcal interaction. In addition, the expression of these genes (by scRNA-seq) was correlated to expression of other genes involved in antifungal activity (**Figures 5** **–** **8**).

## Discussion

With the increasing limitations of current therapies combating cryptococcosis and cryptococcal meningitis, the goal of this study was to explore the initial interaction with *C. neoformans* and lung innate immune cells in order to potentially identify other avenues for intervention. The mounting evidence of the heterogeneity of these immune cells led us to investigate subsets of macrophages and dendritic cells (DCs) that provide most of the early protection from this disease. Our first objective was to identify these subsets within the human lung. We used bronchoalveolar lavage (BAL) fluid from human lungs and examined the contents for the presence of each subset using flow cytometry. Not only did we locate each of the subsets as previously reported (70), but also identified their interaction with *C. neoformans*. Our data show that each of the six subsets (three macrophage and three DC) were able to interact with the fungus in different capacities. Our results were similar to those in previous studies where there was relatively low uptake of certain bacterial pathogens by different subsets at low MOIs (70). These findings were also similar to the high association of cryptococcal cells with pulmonary phagocyte subsets in the mouse model (69).

In investigating the killing capacities of these subsets, we examined their intracellular morphologies and correlated that to cryptococcal fate, as previously done in both *in vitro* human and mouse models (73). In our studies, we found that two macrophage subsets were completely unable to kill *C. neoformans*. Thus, phagocytosis by different macrophage/DC subsets can result in different specific fates for the fungus. In a mouse model, there were more subtle differences in interaction with *C. neoformans* by the phagocytic subsets, with the most significant killing ability exhibited in the Ly6c^-^ monocyte-like macrophages, and the most significant intracellular growth in the CD11b^+^ DCs (69). In our data, we found that AMs and the DC subsets had antifungal activity, but the CD14^+^ and CD14^-^ macrophages did not have antifungal activity. *Mycobacterium tuberculosis* (Mtb) is another pathogen with a similar disease presentation as *C. neoformans* in their ability to evade the host immune system intracellularly by promoting a Th2 response (91). Unlike this study, against Mtb, alveolar macrophages were permissive for bacterial replication and allowed rampant growth (91), whereas interstitial macrophages had antibacterial activity (67).

In identifying differential killing capacities between the subsets of phagocytic APCs, the question that now arises is the inherent molecular differences and transcriptomes between the groups. Due to these cell’s short lifespan outside the human body, we were unable to separate the subsets into discrete populations and conduct a series of focused experiments on individual subsets. Therefore, we performed single cell RNA sequencing (scRNA-seq) on the entire population to obtain the resolution needed that is required in comparing cells or groups of cells to one another. Using subset-specific genes identified previously (70), we found that subsets did not correlate with computational clustering, and we were unable to identify these discrete subsets in our samples. Despite being heterogeneous, these phagocytic APCs are still closely related to one another, and cellular processes and interactions with pathogens such as *C. neoformans* can have a more pronounced effect on their different interactions with pathogenic microbes.

To understand these effects that fungal interaction may have on the population, we performed a trajectory analysis do discover how the clusters are related to one another. Differences in mitochondrial functions and lipid metabolism coupled with immune recognition create the first divergence of the cell populations. Two main transcriptomic groups occurred, the first displaying anticryptococcal characteristics with increases of scavenger receptors to detect fungal products, phagosome maturation which kills the pathogen, and finally antigen presentation to continue the effort against the infection. These groups also displayed a higher-level gene expression relating to fatty acid metabolism (*FABP4*) than those in the second transcriptomic group. This differs from protective immune responses from Mtb, a pathogen with a similar disease manifestation, in that for Mtb, macrophages use glycolytic processes in an effort to kill the bacteria while permissive alveolar macrophages allow growth while using fatty acids and iron (68). However, our results do concur with the data from the cryptococcal mouse model where we observed similar gene expression associated with antifungal responses (genes involved in antigen presentation) *vs.* permissive responses (mitochondrial genes) (69).

The trajectory analysis also informs us of a secondary path the anticryptococcal cells are taking: one that follows an increase in TNF-α transcription from top to bottom. Despite a single incubation period, individual immune cells do not interact with each fungal cell at the same time for the same amount of time but rather at different time throughout a dynamic process. The trajectory analysis can inform us of these dynamics and order the direction by way of pseudotimes (92). Under that prism, we can see a progression of anticryptococcal activity ranging from “naïve” in the top clusters going through killing in the middle clusters and finally ending up at cessation. As first line defenders, macrophages and DCs must recognize, phagocytose, and kill the pathogen until proper adaptive immunity can emerge to control the pathogen. In the upper (early) clusters we see an increase in scavenger receptors. This is in line with other work showing that the scavenger receptor MARCO organizes and enhances antifungal activity during early cryptococcal infections (93). Additionally, the upper clusters demonstrate increases in both lysosomal indicators, cryptococcal killing molecules, and antigen presentation pathways. Inferring, that lower (later) clusters represent more mature pathogen-host cell interactions, these processes give way to more cytokine signaling events along with increased production of TNF-α and some of its downstream products which, again, potentiates cryptococcal defenses (81).

The next step in investigating interactions between pulmonary phagocytic APCs and *C. neoformans* is to understand the extreme early time points where the host cell first reacts to the invading pathogen. Using TNF-α as the indicator for this initial recognition, we examined the differences between early clusters #1 and #9 which have differential TNF-α expression. Before the induction of this cytokine, the immune cell has most of the molecules necessary for the detection and destruction of the fungus. As TNF-α becomes upregulated, there is a large changing of the cell’s proteome as evidenced by the need to first change its protein production and increase its ribosomes. This is also accompanied by increases in fatty acid metabolism through FABP4 which then acts as a link between this metabolism and inflammation (94). Through the scavenger receptors MARCO and CXCL16, intracellular lipid concentration is increased (possibly creating foam cells) which triggers FABP4 (95, 96). This then activates inflammation through both the IKK-NF-κβ and JNK-AP-1 pathways (97, 98). Among the inflammatory cytokines produced from these pathways include CCL3, CXCL8, IL-12, and IL-23 which each contribute to the control of a cryptococcal infection through recruitment of other immune cells or enhancing a Th1 or Th17 adaptive immune response (30).

**Figure 10:**
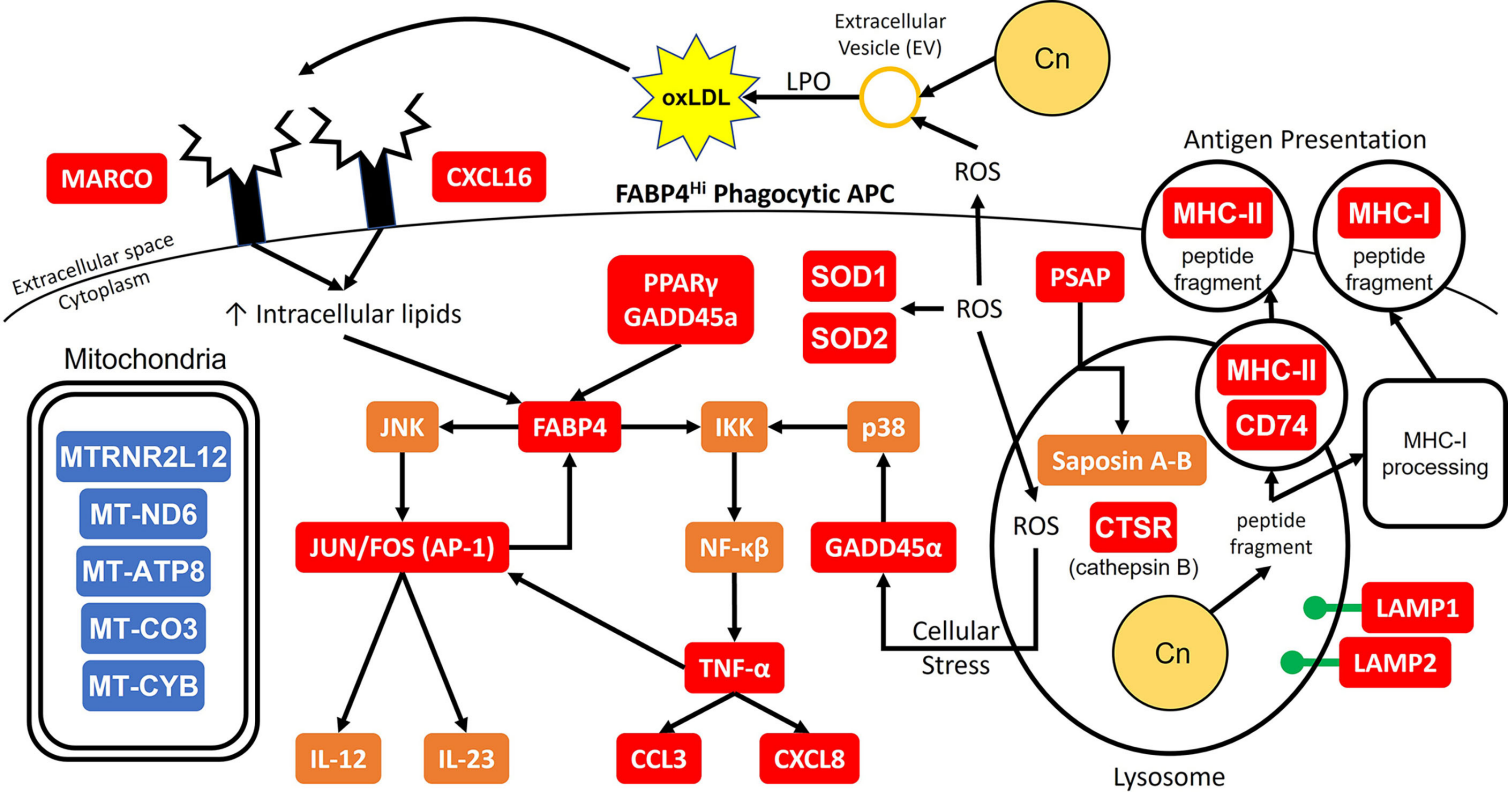
Model for Early Cryptococcal Interactions with Phagocytic APCs. Interactions of *C. neoformans* (Cn) with human pulmonary phagocytic APCs with high expression of FABP4 leads to downstream activation of genes including NF-κβ and AP-1 and phagosome maturation, and down-regulation of many mitochondrial genes. Red and blue boxes indicate identified up- and down-regulated genes, respectively. Orange boxes indicate predicted activation.

Combining all these data, a proposed model of the initial interaction between *C. neoformans* and antifungal subsets of phagocytic APCs is displayed in **Figure 10**. In our model, the cells with the highest propensity for cryptococcal killing are those that have a higher innate usage of fatty acid metabolism (FABP4^Hi^). Interactions begin with recognition of the pathogen, which may happen through scavenger receptors MARCO and CXCL16 which bind to oxidized low-density lipoprotein (oxLDL) (95). The source of these lipids is unclear, one possible supply is from lung surfactant that is highly abundant and necessary for proper pulmonary function (99). However, in our studies this is unlikely as these studies are *ex vivo* and do not contain any external lung surfactant, though residual phagocytosed surfactant could serve as a source. Another possible source could be from the pathogen itself while it produces extracellular vesicles (EVs) (100), which are in turn oxidized by reactive oxygen species (ROS) released by the host cell in a process known as lipid peroxidation (LPO) (101). Whatever the source, these lipid products are taken up by the cell to increase its intracellular lipid concentration further activating FABP4. FABP4 can then initiate inflammation through either the JNK-AP-1 or IKK-NF-κβ pathways (94). Additionally, FABP4 can directly regulate mitochondrial function (102). Meanwhile during these early events, phagocytosis occurs with subsequent endosomal processing bringing the pathogen into a phagolysosomal compartment to begin destruction of the fungus. In this process, lysosome markers LAMP1/2 are increased along with other mediators such as ROS and cathepsin B (*CTSB*) as it combats *C. neoformans* (73, 103). Additionally, the cell will deploy saposins (from the precursor protein prosaposin [*PSAP*]) to degrade sphingolipids used by the fungus to facilitate intracellular growth (104, 105). Destroying the pathogen results in peptide fragments which can be used in antigen presentation through either the MHC-I or MHC-II pathways (106, 107). These actions put considerable stress on the host cell. This is sensed by GADD45α which activates NF-κβ through p38. Along with PPARγ, GADD45α can also increase the expression of FABP4 (108, 109). Finally, the NF-κβ pathway will increase the expression of TNF-α and its downstream mediators such as CCL3 and CXCL8 while the AP-1 pathway will upregulate the Th1 and Th17 mediators IL-12 and IL-23 (110–112). There are additional feedback and cross loops in this model which include JUN/FOS which feedback into FABP4 and TNF-α cross-feeding into AP-1 (98, 113).

Being one of the first studies conducted on human phagocytic APCs and their initial interactions with *C. neoformans* with single cell resolution, we hope to provide some potential avenues for combating this pathogen. We were unable to determine the exact reason for differences in cryptococcal killing, however, our transcriptional data show that fatty acid metabolism is closely related to anticryptococcal activities by providing signals that confer a protective phenotype. Furthermore, specific proteins targets have been identified and their place in a model of cryptococcal infection must be thoroughly inspected. Identifying these protein targets and their abundance within the cells at different stages of interactions is important as the association between transcript level and protein amounts may not always strictly correlate, and may not carry a biological meaning (114). Ongoing studies in our lab are examining this relationship closely to determine the causal effects these networks have on one another. We are conducting studies to validate the gene expression data from the single-cell RNA-seq, examining metabolic differences in antifungal cells *vs.* permissive cells, and silencing upregulated genes in order to determine their role(s) in antifungal activity. The ultimate question is whether the expression of these genes and corresponding proteins confers a differential phenotype on the outcome of intracellular cryptococcal cells. In addition, we are interested in the networks that are activated and mechanisms used in successful antifungal host cells. The present study lays the foundation for future work to examine these areas and discover new treatments for pulmonary cryptococcosis.

## Data availability statement

The raw data are deposited into the Gene Expression Omnibus database, GEO accession number is GSE216963.

## Ethics statement

Ethical review and approval was not required for the study on human participants in accordance with the local legislation and institutional requirements. Written informed consent for participation was not required for this study in accordance with the national legislation and the institutional requirements.

## Author contributions

KW designed the study, performed analyses, interpreted study results, and participated in drafting and editing of the manuscript. JB, VP, and JM collected and processed de-identified clinical specimens. BN and CD assisted in study design, performed experiments and statistical analyses, and participated in interpretation of results. BN and CD performed the experiments. BN, RS, KJ, and KW analyzed the data KW, BN, JM, and KJ wrote and edited the manuscript. KW planned and supervised data analysis and interpretation, supervised the study, and revised the manuscript. All authors contributed to the article and approved the submitted version.

## Funding

This work was supported by a research grant 1P20GM134973-01 (KW, JM) from the National Institute of General Medical Sciences of the National Institutes of Health (NIH), and a research grant I01 BX001937 from the Merit Review Program of the Department of Veterans Affairs (JM). The funders had no role in study design, data collection or analysis, decision to publish, or preparation of the manuscript.

## Acknowledgments

We thank the Institutional Research Core Facility at the University of Oklahoma Health Science Center (OUHSC), Oklahoma City, OK for the use of the Core Facility which provided imaging flow cytometry (Flow Cytometry and Imaging Core) and RNA sequencing (Sequencing and Genomic Core) services. We would also like to thank the following people for their support and technical expertise: Dr. Lauren Zenewicz (OUHSC), Jenny Gipson (OUHSC), and Dr. James Henthorn (OUHSC).

## Conflict of interest

The authors declare that the research was conducted in the absence of any commercial or financial relationships that could be construed as a potential conflict of interest.

## Publisher’s note

All claims expressed in this article are solely those of the authors and do not necessarily represent those of their affiliated organizations, or those of the publisher, the editors and the reviewers. Any product that may be evaluated in this article, or claim that may be made by its manufacturer, is not guaranteed or endorsed by the publisher.

## Supplementary material

Supplementary Figure 1 Representative Image of CrAg^®^ LFA Results. Prior to processing, human BAL samples were tested for active cryptococcal infection by the commercially available CrAg^®^ LFA kit. From left to right are shown representative images of a negative control, sample test result (#1), and a positive control. One line indicates a negative result while two lines indicate a positive result. Two replicate tests were performed on 12 samples (n=12).

Supplementary Figure 2 Representative Gating Scheme for Identification of Phagocytic APC Subsets during Flow Cytometry. The gating strategy used for identification of the 6 phagocytic APC subsets is shown. Red boxes indicate gates and red arrows point towards new plots from previous gates. Combination of positive and negative gating was used to identify subsets. Bottom six plots display total population for each of the six subsets with their respective uptake of mCherry expressing *C. neoformans*. Controls included unstained and single-color samples for color compensation as well as isotype control for false-positive identification.

Supplementary Figure 3 Representative Gating Scheme for Identification of Phagocytic APC Subsets during Imaging Flow Cytometry. The gating strategy used for identification of the 6 phagocytic APC subsets during imaging flow cytometry is shown. Colored boxes indicate gates and arrows point towards new plots from previous gates. **(A)** Scheme used with imaging flow panel #1 for the identification of alveolar macrophages. **(B)** Scheme used with imaging flow panel #2 for the identification of all other subsets. Combination of positive and negative gating was used to identify subsets along with internalization mask for internalized cryptococcal cells. Controls included single-color samples for color compensation.

Supplementary Figure 4 Identification of Phagocytic APC Subsets by Subset Specific Upregulated Genes. Processed human BAL cells were enriched for phagocytic APCs and incubated with **(G–L)** or without **(A–F)** *C. neoformans* strain H99 for 2h and then processed for scRNA-seq. Phagocytic APC subsets were identified using upregulated genes *ACACB* **(A/G)**, *ADAMTS2* **(B/H)**, *DPP4* **(C/I)**, *CD207* **(D/J)**, *ANTXR1* **(E/K)**, and *CNKSR3* **(F/L)** for AMs, CD14^+^CD1c^-^ macs, CD14^-^CD1c^-^ macs, CD207^+^ DCs, CD14^+^CD1c^+^ DCs, and CD14^-^CD1c^+^ DCs, respectively. Data shown are transcriptional regulation of shown genes for 18,958 (naïve) and 22,344 (infected) total cells for three individual experiments for each cohort (n=3).

Supplementary Figure 5 No Differential Macrophage Activation or Th1/Th2 Bias among the Cells. Processed human BAL cells were enriched for phagocytic APCs and incubated with *C. neoformans* strain H99 for 2h and then processed for scRNA-seq. Immune cells from the infected cohort were examined for markers for macrophage M1 or Th1-type cytokine genes (*IFN-γ*, *IL-12A*, *IL-12B*, *NOS2*) **(A–D)** and M2 or Th2-type cytokine genes (*IL-4*, *IL-10*, *IL-13*, *ARG1*) **(E–H)** activation. The cells experienced little to no upregulation for any of the genes. Data shown are transcriptional regulation of shown genes for 22,344 total cells for three individual experiments (n=3).

Supplementary Figure 6 Representative Gating Scheme for Correlation of TNF-α Markers and Cryptococcal Morphology. The gating strategy used for identification of cryptococcal morphology as they correlate to TNF-α markers during imaging flow cytometry is shown. Colored boxes indicate gates and arrows point towards new plots from previous gates. **(A)** Scheme used with TNF-α panel #1 for correlation with markers CXCL8 and CCL3. **(B)** Scheme used with TNF-α panel #2 for correlation with markers TNF-α and IL-1β. Combination of positive and negative gating was used to identify individual markers and cryptococcal interaction. Controls included single-color samples for color compensation.

Supplementary Figure 7 Representative Images of TNF-α Markers and Cryptococcal Morphology. Processed human BAL cells were enriched for phagocytic APCs and incubated with the fluorescent mCherry expressing *C. neoformans* strain JLCN920 for 2h and then stained with fluorescent antibodies for imaging flow cytometric analysis. Representative images of internalized *C. neoformans* to determine fate of the fungus. Budding and round fungal cells were considered as living while condensed and debris were deemed as dead cells. **(A)** Representative images for TNF-α and IL-1β. **(B)** Representative images for CXCL8 and CCL3. Combination of positive and negative gating was used to identify individual markers and cryptococcal interaction. Controls included single-color samples for color compensation.

Supplementary Figure 8 TNF-α Signaling Is Not an Indicator for Cryptococcal Fate. Processed human BAL cells were enriched for phagocytic APCs and incubated with fluorescent mCherry expressing *C. neoformans* strain JLCN920 for 2h and then stained with fluorescent antibodies for imaging flow cytometric analysis. Analysis revealed no significant differences between living and killed cryptococcal cells for cytokines TNF-α **(A)**, IL-1β **(B)**, CXCL8 **(C)**, or CCL3 **(D)**. Examination of fluorescent strengths for cryptococcal intensities (y-axis) against TNF-α **(E)**, IL-1β **(F)**, CXCL8 **(G)**, or CCL3 **(H)** (x-axis) exhibited a strong positive correlation. Data shown for panels A-D are mean fluorescent intensities (MFI) of individual viable CD45^+^ phagocytic APCs from three individual experiments (n=3). Panels **(E–H)** show pooled data from the 3 experiments in panels **(A–D)** of the same cells. Trend line is represented by the red line. Two-tailed *t*-tests were performed to compare pairs of MFIs with notation “ns” to represent no significance. Linear regression was performed to compare correlations of cryptococcal interaction with TNF- α markers. Slopes of all interactions were significantly non-zero *(p* < 0.0001).
