# Supplementary material for: Protective interaction of human phagocytic APC subsets with Cryptococcus neoformans induces genes associated with metabolism and antigen presentation
Source: Front Immunol. 2022 Nov 15;13:1054477. doi: 10.3389/fimmu.2022.1054477 (PMC9709479; doi:10.3389/fimmu.2022.1054477)
Supplement: Supplementary file 1 [file Table_1.docx]

**Table S1 ǀ Lung Cell Donor Demographics and Cell Yield.** Demographics for patient samples used in this study.

| **Donor #** | **Age** | **^a^ Race** | **Gender** | **^b^ Peak PaO2/FiO2** | **^b^ Final PaO2/FiO2** | **Chest X-Ray Results** | **Cell Yield** |
| --- | --- | --- | --- | --- | --- | --- | --- |
| 1 | 29 | AA | Male | 438 | 438 | Normal except for scattered areas of nodularity within right lung suggestive of bronchiolitis. | 5.62 x 10^9^ |
| 3 | 57 | AA | Male | 540 | 383 | Normal except for stable left basilar infiltrate with layering pleural effusion and stable right mild basilar infiltrate. | 4.61 x 10^9^ |
| 4 | 35 | AA | Male | 363 | 262 | Normal except for stable right pleural effusion and volume loss in right lung base. | 6.02 x 10^9^ |
| 5 | 52 | C | Female | 244 | 139 | Normal except for persistent pneumomediastinum, and persistent soft tissue gas in the neck and left chest wall. | 3.36 x 10^9^ |
| 6 | 34 | C | Male | 329 | 321 | Normal except for mild bilateral parahilar and basilar infiltrates. | 7.30 x 10^9^ |
| 7 | 48 | C | Male | 344 | 243 | Normal except for increasing retro cardiac density, blunting in the region of the left costophrenic angle, and exclusion of the right costophrenic angle. | 7.01 x 10^9^ |
| 8 | 28 | C | Female | 283 | 338 | Normal except for bibasilar pulmonary opacities. Possible small pleural effusion. | 1.60 x 10^9^ |
| 9 | 54 | AA | Male | 410 | 410 | Normal except for left lower lobe atelectasis, with right basilar opacity suggesting atelectasis | 1.24 x 10^9^ |
| 10 | 43 | C | Female | 404 | 328 | Normal except for bibasilar airspace infiltrates. | 1.80 x 10^9^ |
| 11 | 48 | C | Female | 427 | 416 | Normal except for a few hazy low lung opacities, indicative of layering effusions, atelectasis, and/or airspace disease. | 1.92 x 10^9^ |
| 12 | 31 | C | Female | 434 | 211 | Normal appearance. Lungs are expanded and clear. No pneumothorax or pleural effusion. | 8.84 x 10^9^ |
| 13 | 53 | C | Female | 390 | 390 | Normal except for left basilar opacities and small left pleural effusion suspected. | 4.93 x 10^9^ |

^a^ AA, C = African American, Caucasian

^b^ PaO2/FiO2 = partial pressure arterial oxygen / fraction of inspired oxygen.
