# Supplementary material for: Protective interaction of human phagocytic APC subsets with Cryptococcus neoformans induces genes associated with metabolism and antigen presentation
Source: Front Immunol. 2022 Nov 15;13:1054477. doi: 10.3389/fimmu.2022.1054477 (PMC9709479; doi:10.3389/fimmu.2022.1054477)
Supplement: Supplementary file 2 [file Table_2.docx]

**Table S2 ǀ Antibodies and viability dyes used for flow cytometry.** List of all antibodies and conjugated fluorophores used in each flow cytometry panel

| **Marker** | **Purpose** | **Fluorophore** | **Clone** | **Company** | **Assay used in:** | | | | |
| --- | --- | --- | --- | --- | --- | --- | --- | --- | --- |
|  |  |  |  |  | Standard flow panel | Imaging flow panel #1 | Imaging flow panel #2 | TNF-α panel #1 | TNF-α panel #2 |
| Fixable Aqua | Viability | Fixable Aqua | N/A | ThermoFisher | + | - | - | - | - |
| Fixable Near IR | Viability | Fixable Near IR | N/A | ThermoFisher | - | - | - | + | + |
| CD45 | Leukocyte | BV605 | 2D1 | BioLegend | + | - | - | - | - |
| CD45 | Leukocyte | PE | 2D1 | Invitrogen | - | + | - | - | - |
| CD45 | Leukocyte | PerCP efluor 710 | HI30 | Invitrogen | - | - | + | + | + |
| Lineage cocktail  (CD3, CD19, CD20, CD56) | Lymphocyte  lineage | APC | UCHT1, HIB19,  2H7, 5.1H11 | BioLegend | + | - | - | - | - |
| Calcofluor white | External fungi | Calcofluor white | N/A | ThermoFisher | + | - | - | - | - |
| CD207 | Subset | PE | 10E2 | BioLegend | + | - | - | - | - |
| CD11c | Subset | PerCP efluor 710 | 3.9 | Invitrogen | + | + | - | - | - |
| CD1c (BDCA-1) | Subset | PE-Vio 770 | AD5-8E7 | Miltenyi | + | - | - | - | - |
| CD14 | Subset | APC efluor 780 | 61D3 | Invitrogen | + | - | - | - | - |
| HLA-DR | Subset | BV650 | G46-6 | BD Biosciences | + | - | - | - | - |
| CD123 | Subset | BV785 | 6H6 | BioLegend | + | - | - | - | - |
| CD14 | Subset | AF405 | RPA-M1 | Novus | - | + | + | - | - |
| CD1c | Subset | FITC | L161 | Invitrogen | - | + | + | - | - |
| CD207 | Subset | PE | 10E2 | BioLegend | - | - | + | - | - |
| CXCL8/IL-8 | TNF-α marker | FITC | NAP II | Invitrogen | - | - | - | + | - |
| CCL3/MIP-1α | TNF-α marker | PE | CR3M | Invitrogen | - | - | - | + | - |
| IL-1β | TNF-α marker | FITC | CRM56 | Invitrogen | - | - | - | - | + |
| TNF-α | TNF-α marker | PE | Mab11 | Invitrogen | - | - | - | - | + |
