# Supplementary material for: Protective interaction of human phagocytic APC subsets with Cryptococcus neoformans induces genes associated with metabolism and antigen presentation
Source: Front Immunol. 2022 Nov 15;13:1054477. doi: 10.3389/fimmu.2022.1054477 (PMC9709479; doi:10.3389/fimmu.2022.1054477)
Supplement: Supplementary file 3 [file Table_3.docx]

**Table S3 ǀ Active cryptococcal detection results.** Patient sample results for CrAg® LFA

| **Sample Number** | **CrAg® LFA Results** |
| --- | --- |
| 1 | Negative (-) |
| 2 | Negative (-) |
| 3 | Negative (-) |
| 4 | Negative (-) |
| 5 | Negative (-) |
| 6 | Negative (-) |
| 7 | Negative (-) |
| 8 | Negative (-) |
| 9 | Negative (-) |
| 10 | Negative (-) |
| 11 | Negative (-) |
| 12 | Negative (-) |
