# Supplementary figures and images for: Protective interaction of human phagocytic APC subsets with Cryptococcus neoformans induces genes associated with metabolism and antigen presentation
Source: Front Immunol. 2022 Nov 15;13:1054477. doi: 10.3389/fimmu.2022.1054477 (PMC9709479; doi:10.3389/fimmu.2022.1054477)

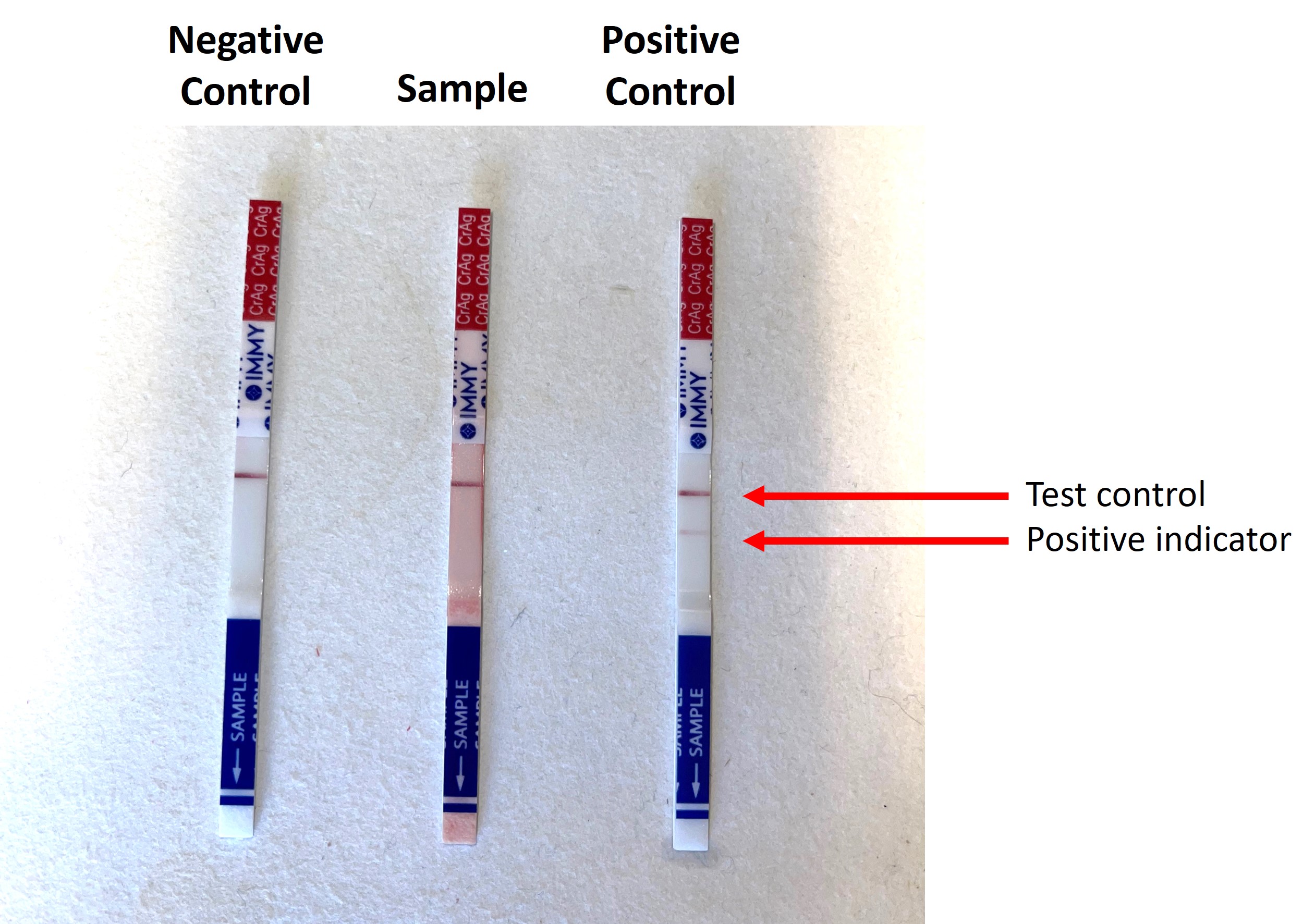

Supplement: Supplementary Figure 1 — Representative Image of CrAg® LFA Results. Prior to processing, human BAL samples were tested for active cryptococcal infection by the commercially available CrAg® LFA kit. From left to right are shown representative images of a negative control, sample test result (#1), and a positive control. One line indicates a negative result while two lines indicate a positive result. Two replicate tests were performed on 12 samples (n=12). [file Image_1.jpeg]

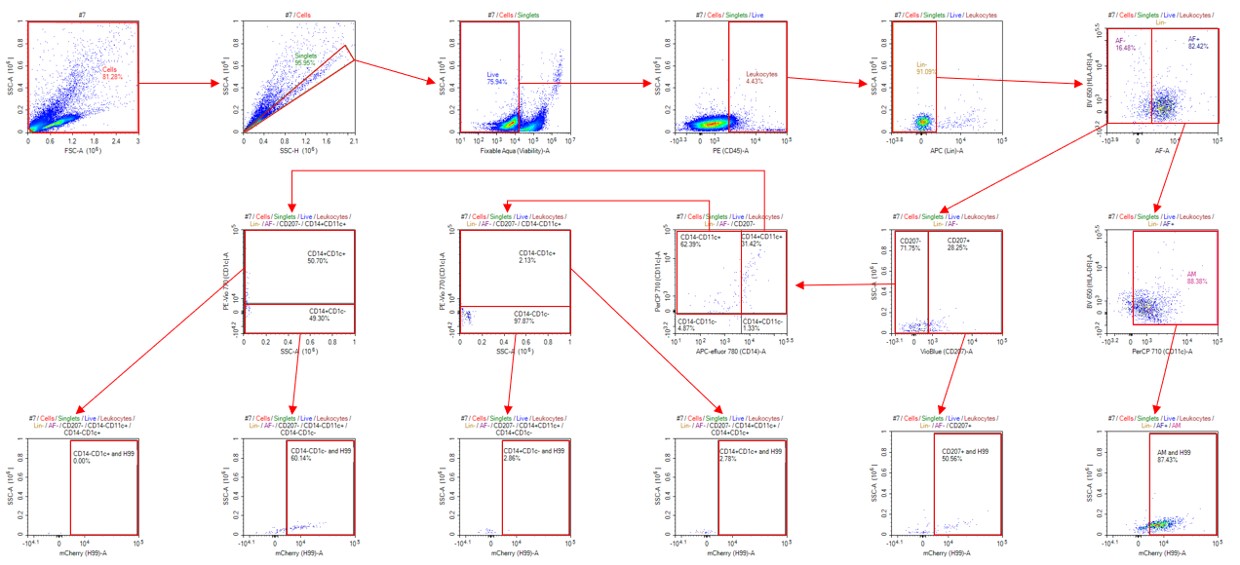

Supplement: Supplementary Figure 2 — Representative Gating Scheme for Identification of Phagocytic APC Subsets during Flow Cytometry. The gating strategy used for identification of the 6 phagocytic APC subsets is shown. Red boxes indicate gates and red arrows point towards new plots from previous gates. Combination of positive and negative gating was used to identify subsets. Bottom six plots display total population for each of the six subsets with their respective uptake of mCherry expressing C. neoformans. Controls included unstained and single-color samples for color compensation as well as isotype control for false-positive identification. [file Image_2.jpeg]

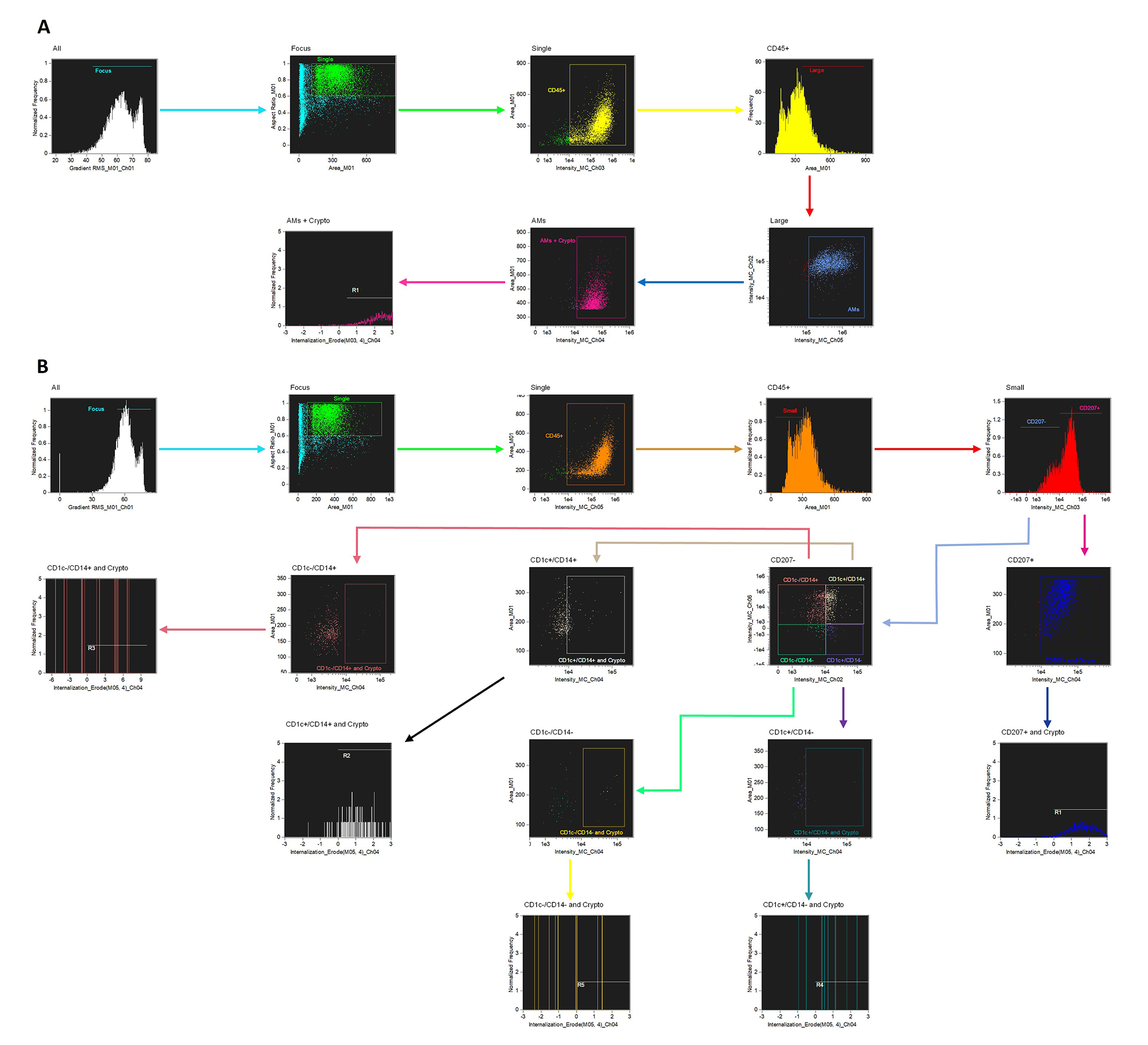

Supplement: Supplementary Figure 3 — Representative Gating Scheme for Identification of Phagocytic APC Subsets during Imaging Flow Cytometry. The gating strategy used for identification of the 6 phagocytic APC subsets during imaging flow cytometry is shown. Colored boxes indicate gates and arrows point towards new plots from previous gates. (A) Scheme used with imaging flow panel #1 for the identification of alveolar macrophages. (B) Scheme used with imaging flow panel #2 for the identification of all other subsets. Combination of positive and negative gating was used to identify subsets along with internalization mask for internalized cryptococcal cells. Controls included single-color samples for color compensation. [file Image_3.jpeg]

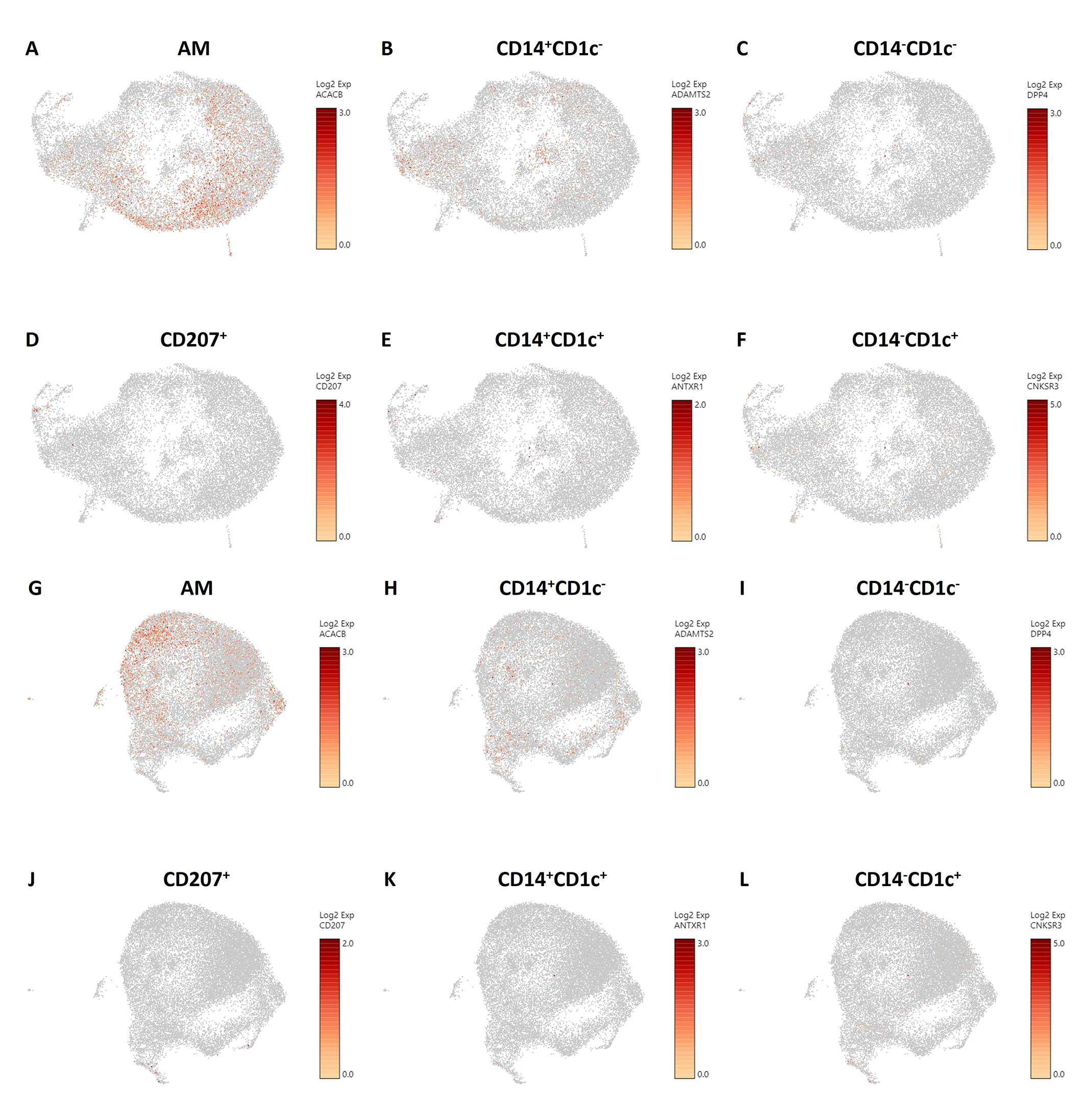

Supplement: Supplementary Figure 4 — Identification of Phagocytic APC Subsets by Subset Specific Upregulated Genes. Processed human BAL cells were enriched for phagocytic APCs and incubated with (G–L) or without (A–F) C. neoformans strain H99 for 2h and then processed for scRNA-seq. Phagocytic APC subsets were identified using upregulated genes ACACB (A/G), ADAMTS2 (B/H), DPP4 (C/I), CD207 (D/J), ANTXR1 (E/K), and CNKSR3 (F/L) for AMs, CD14+CD1c- macs, CD14-CD1c- macs, CD207+ DCs, CD14+CD1c+ DCs, and CD14-CD1c+ DCs, respectively. Data shown are transcriptional regulation of shown genes for 18,958 (naïve) and 22,344 (infected) total cells for three individual experiments for each cohort (n=3). [file Image_4.jpeg]

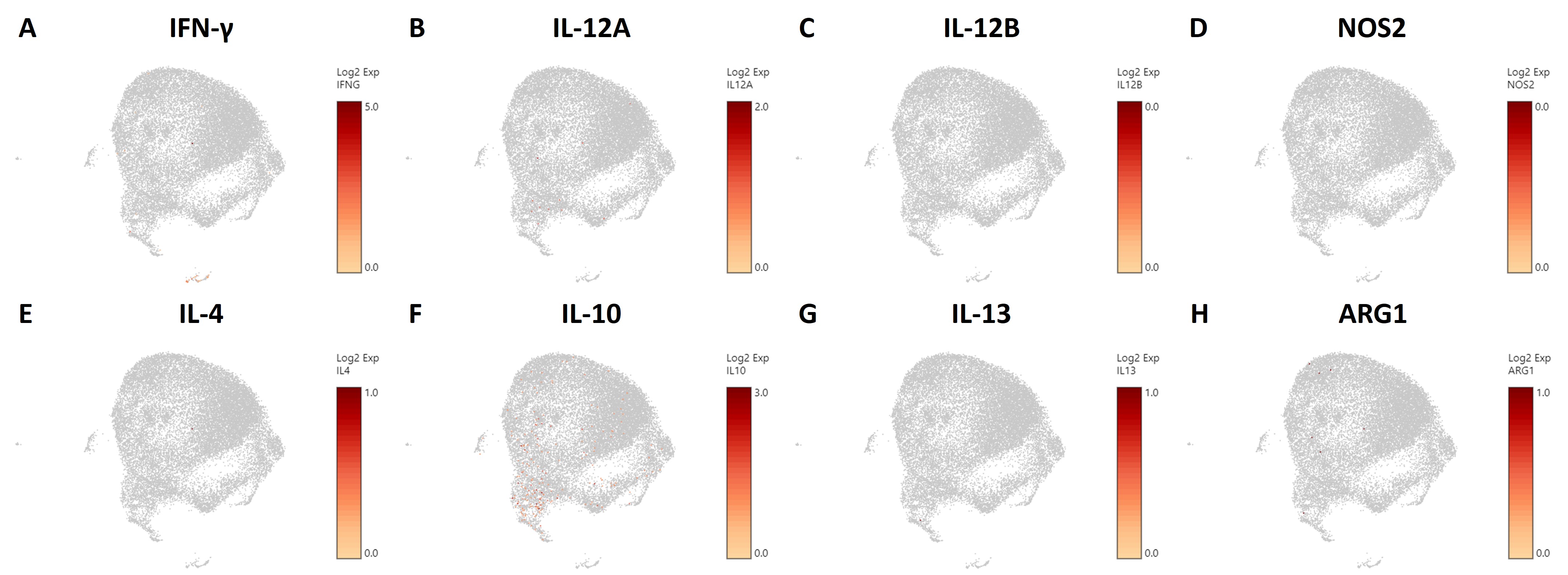

Supplement: Supplementary Figure 5 — No Differential Macrophage Activation or Th1/Th2 Bias among the Cells. Processed human BAL cells were enriched for phagocytic APCs and incubated with C. neoformans strain H99 for 2h and then processed for scRNA-seq. Immune cells from the infected cohort were examined for markers for macrophage M1 or Th1-type cytokine genes (IFN-γ, IL-12A, IL-12B, NOS2) (A–D) and M2 or Th2-type cytokine genes (IL-4, IL-10, IL-13, ARG1) (E–H) activation. The cells experienced little to no upregulation for any of the genes. Data shown are transcriptional regulation of shown genes for 22,344 total cells for three individual experiments (n=3). [file Image_5.jpeg]

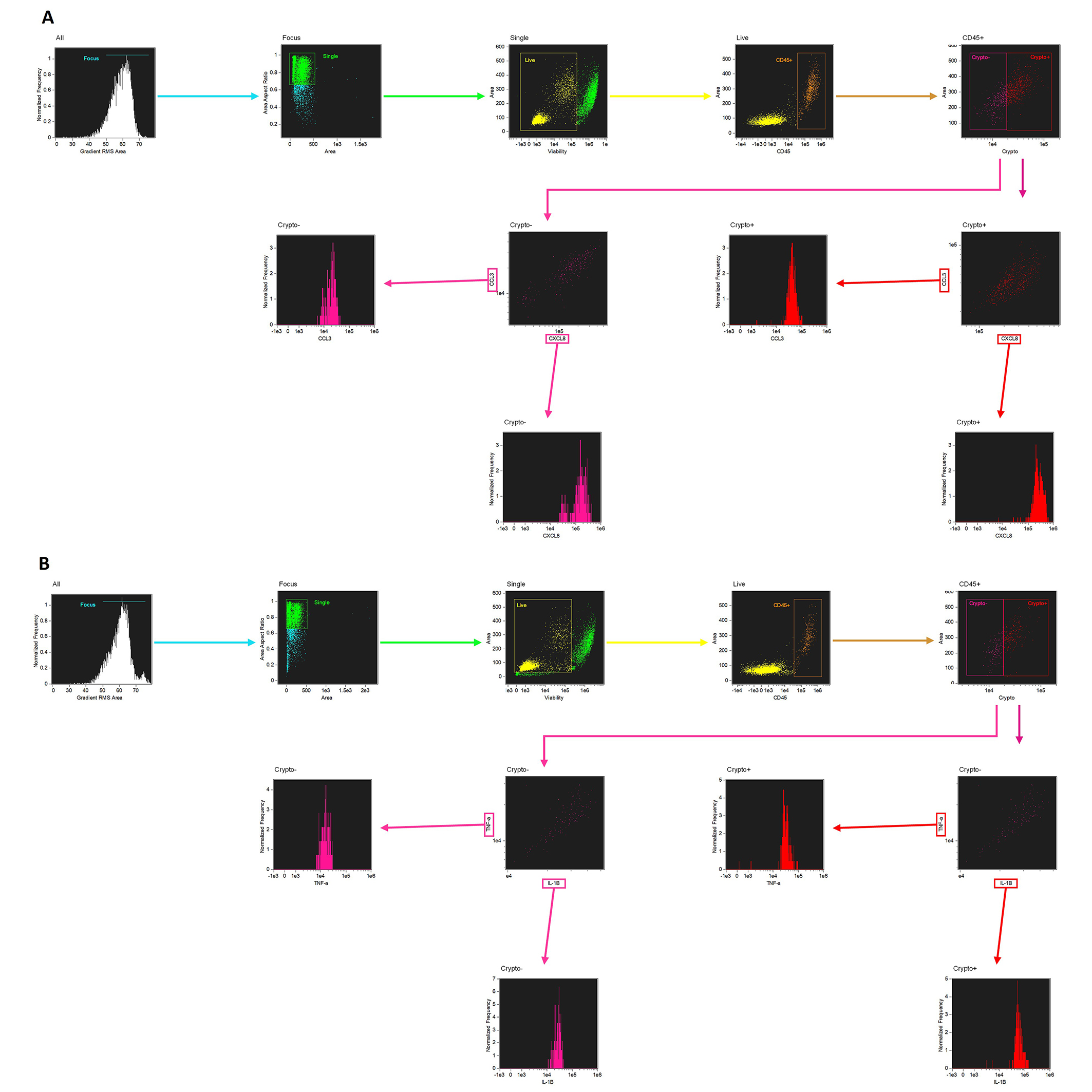

Supplement: Supplementary Figure 6 — Representative Gating Scheme for Correlation of TNF-α Markers and Cryptococcal Morphology. The gating strategy used for identification of cryptococcal morphology as they correlate to TNF-α markers during imaging flow cytometry is shown. Colored boxes indicate gates and arrows point towards new plots from previous gates. (A) Scheme used with TNF-α panel #1 for correlation with markers CXCL8 and CCL3. (B) Scheme used with TNF-α panel #2 for correlation with markers TNF-α and IL-1β. Combination of positive and negative gating was used to identify individual markers and cryptococcal interaction. Controls included single-color samples for color compensation. [file Image_6.jpeg]

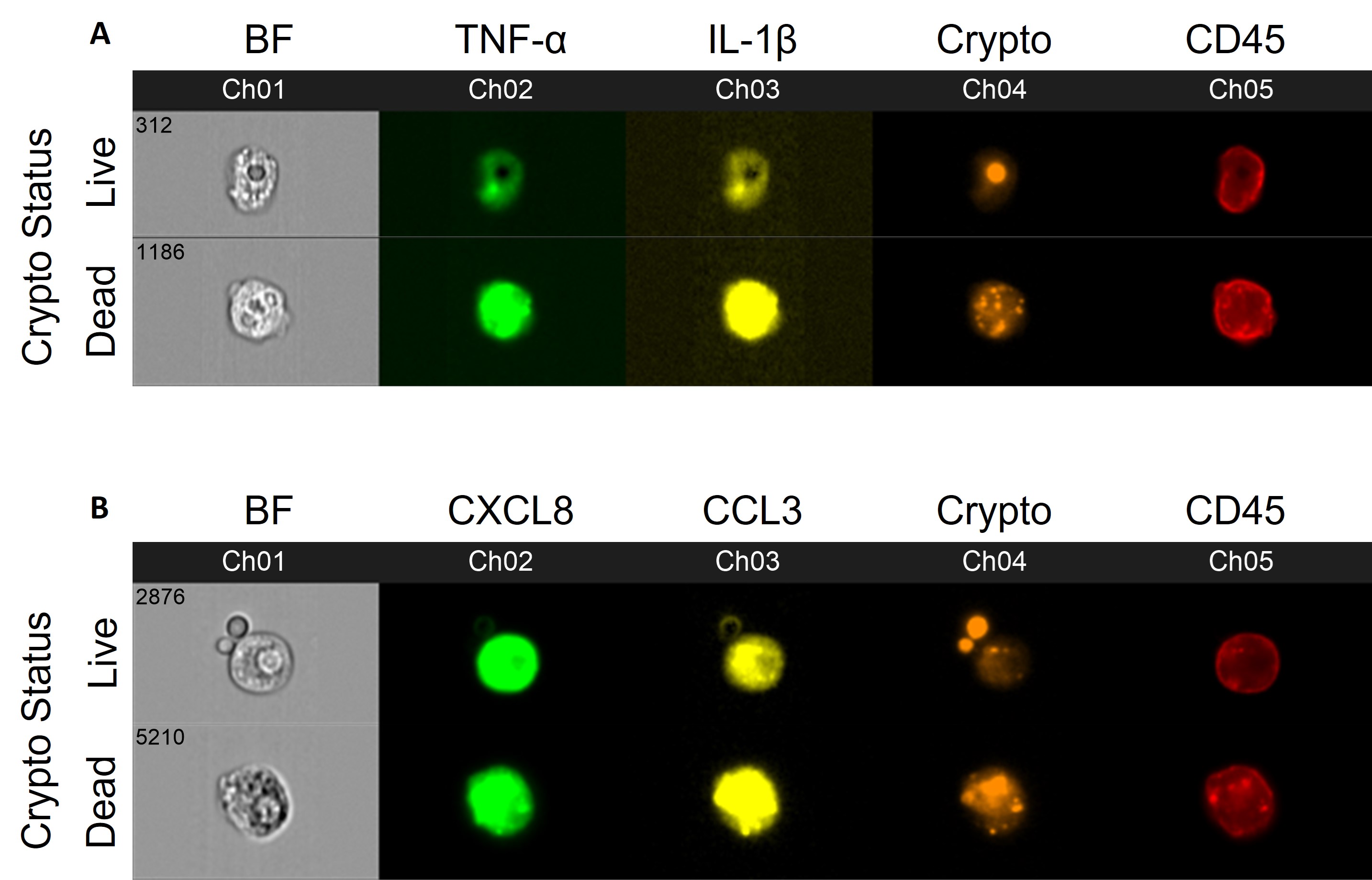

Supplement: Supplementary Figure 7 — Representative Images of TNF-α Markers and Cryptococcal Morphology. Processed human BAL cells were enriched for phagocytic APCs and incubated with the fluorescent mCherry expressing C. neoformans strain JLCN920 for 2h and then stained with fluorescent antibodies for imaging flow cytometric analysis. Representative images of internalized C. neoformans to determine fate of the fungus. Budding and round fungal cells were considered as living while condensed and debris were deemed as dead cells. (A) Representative images for TNF-α and IL-1β. (B) Representative images for CXCL8 and CCL3. Combination of positive and negative gating was used to identify individual markers and cryptococcal interaction. Controls included single-color samples for color compensation. [file Image_7.jpeg]

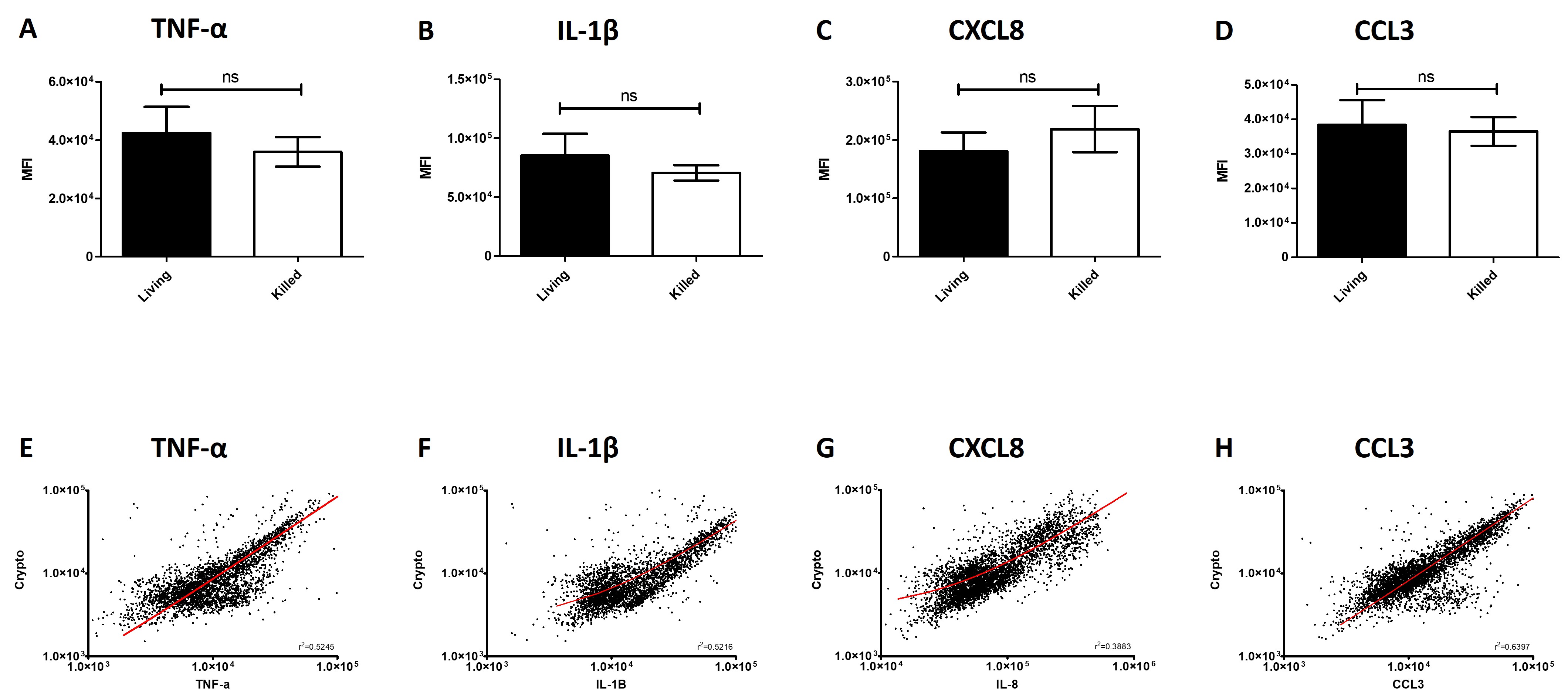

Supplement: Supplementary Figure 8 — TNF-α Signaling Is Not an Indicator for Cryptococcal Fate. Processed human BAL cells were enriched for phagocytic APCs and incubated with fluorescent mCherry expressing C. neoformans strain JLCN920 for 2h and then stained with fluorescent antibodies for imaging flow cytometric analysis. Analysis revealed no significant differences between living and killed cryptococcal cells for cytokines TNF-α (A), IL-1β (B), CXCL8 (C), or CCL3 (D). Examination of fluorescent strengths for cryptococcal intensities (y-axis) against TNF-α (E), IL-1β (F), CXCL8 (G), or CCL3 (H) (x-axis) exhibited a strong positive correlation. Data shown for panels A-D are mean fluorescent intensities (MFI) of individual viable CD45+ phagocytic APCs from three individual experiments (n=3). Panels (E–H) show pooled data from the 3 experiments in panels (A–D) of the same cells. Trend line is represented by the red line. Two-tailed t-tests were performed to compare pairs of MFIs with notation “ns” to represent no significance. Linear regression was performed to compare correlations of cryptococcal interaction with TNF- α markers. Slopes of all interactions were significantly non-zero (p < 0.0001). [file Image_8.jpeg]
